# Scaffold-Mediated Immunoengineering as Innovative Strategy for Tendon Regeneration

**DOI:** 10.3390/cells11020266

**Published:** 2022-01-13

**Authors:** Valentina Russo, Mohammad El Khatib, Giuseppe Prencipe, Adrián Cerveró-Varona, Maria Rita Citeroni, Annunziata Mauro, Paolo Berardinelli, Melisa Faydaver, Arlette A. Haidar-Montes, Maura Turriani, Oriana Di Giacinto, Marcello Raspa, Ferdinando Scavizzi, Fabrizio Bonaventura, Liliana Liverani, Aldo R. Boccaccini, Barbara Barboni

**Affiliations:** 1 Unit of Basic and Applied Sciences, Faculty of Biosciences and Agro-Food and Environmental Technologies, University of Teramo, 64100 Teramo, Italy; vrusso@unite.it (V.R.); melkhatib@unite.it (M.E.K.); acerverovarona@unite.it (A.C.-V.); mrciteroni@unite.it (M.R.C.); amauro@unite.it (A.M.); pberardinelli@unite.it (P.B.); mfaydaver@unite.it (M.F.); aahaidarmontes@unite.it (A.A.H.-M.); mturriani@unite.it (M.T.); odigiacinto@unite.it (O.D.G.); bbarboni@unite.it (B.B.); 2 Institute of Biochemistry and Cellular Biology (IBBC), Council of National Research (CNR), Campus International Development (EMMA-INFRAFRONTIER-IMPC), 00015 Monterotondo Scalo, Italy; mraspa@emma.cnr.it (M.R.); fscavizzi@emma.cnr.it (F.S.); bonaventura@emma.cnr.it (F.B.); 3 Department of Materials Science and Engineering, Institute of Biomaterials, University of Erlangen-Nuremberg, 91058 Erlangen, Germany; lialiana.liverani@fau.de (L.L.); aldo.boccaccini@fau.de (A.R.B.)

**Keywords:** tendon regeneration, tissue engineering, immunoengineering, scaffold, electrospinning, immune response, stem cells, immune cells, biomolecules, mechanotransduction

## Abstract

Tendon injuries are at the frontier of innovative approaches to public health concerns and sectoral policy objectives. Indeed, these injuries remain difficult to manage due to tendon’s poor healing ability ascribable to a hypo-cellularity and low vascularity, leading to the formation of a fibrotic tissue affecting its functionality. Tissue engineering represents a promising solution for the regeneration of damaged tendons with the aim to stimulate tissue regeneration or to produce functional implantable biomaterials. However, any technological advancement must take into consideration the role of the immune system in tissue regeneration and the potential of biomaterial scaffolds to control the immune signaling, creating a pro-regenerative environment. In this context, immunoengineering has emerged as a new discipline, developing innovative strategies for tendon injuries. It aims at designing scaffolds, in combination with engineered bioactive molecules and/or stem cells, able to modulate the interaction between the transplanted biomaterial-scaffold and the host tissue allowing a pro-regenerative immune response, therefore hindering fibrosis occurrence at the injury site and guiding tendon regeneration. Thus, this review is aimed at giving an overview on the role exerted from different tissue engineering actors in leading immunoregeneration by crosstalking with stem and immune cells to generate new paradigms in designing regenerative medicine approaches for tendon injuries.

## 1. Introduction

Tendinopathies are among the most difficult orthopedic injuries to be managed, which in turn imply the development of advanced approaches to fulfill public health challenges and sectoral policy objectives. They represent around 30% of musculoskeletal disorders with multiple risk factors recognized, including overuse, aging, and metabolic diseases (i.e., obesity and diabetes) [1,2]. The associated socioeconomic burden is over EUR 180 billion in the USA and EU, with a forecast of +25% over the next five years as a consequence of the absence of an efficacious therapeutic solution and variations in life expectancy, lifestyle and working conditions [3]. In veterinary medicine around 46% of racehorses are affected by tendinopathy that in turn reduces their performance, generating a negative economic impact of EUR 400 billion worldwide [4].

Tendinopathies are normally characterized by reduced mobility associated with chronic tendon pain and a consequent impairment in the movements that compromises everyday life. In humans, the Achilles, rotator cuff, patellar and forearm flexor and extensor tendons are the most vulnerable [3], while the superficial digital flexor tendon (SDFT) is the most affected in athletic horses and Achilles tendons in dogs [5]. Unfortunately, spontaneous tendon healing is ineffective due to the tissue low cellularity and hypo-vascularity [6]. As a consequence, any tissue damage is only partially repaired with a disorganized extracellular matrix (ECM) production/fibrotic tissue, with reduced biomechanical properties [1,2]. Indeed, inflammation in tendinopathy can persist through key inflammatory mediators which contribute to disrupting the tissue ECM [7]. In detail, the increasing number of inflammatory cells after tendon injury leads to an imbalance between pro-inflammatory factors, provoking the degradation of ECM [8]. Infiltrating immune cells provoke inflammation due to the activation of inflammatory mediators’ pathways, comprising cytokines, e.g., tumor necrosis factor-α (TNF-α), interleukin-1β (IL-1β), IL-6, and prostaglandins including prostaglandin E2 (PGE_2_), hence promoting pro-inflammatory macrophage (M1) and T cell activity. The released inflammatory cytokines exhibit different roles, including the upregulation of vascular endothelial growth factor (VEGF) synthesis together with the enhancement of the production of metalloproteases (MMPs), such as MMP-1, MMP-3, MMP-8, MMP-9, and MMP-13, implicated in inducing matrix destruction [8]. Tenocytes play an accountable role in tissue remodeling and repair due to the presence of immune receptors on their cell surface. Upon activation toward an inflammatory phenotype, tenocytes start secreting inflammatory mediators in both autocrine or paracrine manner that can modulate the inflammatory response and ECM remodeling after injury [9]. Thus, to progress in tendon healing, it is crucial to turn off the inflammatory process and modulate the tendon immune-sensing compartment [10,11]. These evidence on the modulation of inflammation in the resolution of tendinopathies could represent at the same time a novel therapeutic strategy. If inflammation would persist in damaged tendons, possible ulterior damage and degenerative states might lead to chronic tendon injuries which most of the time result in tendon ruptures [12]. Over the last few decades, researchers have been developing new strategies based on tissue engineering (TE) approaches as an alternative solution to the conventional treatments for tendon ruptures [13]. That being said, to date there is no available therapeutic strategy able to restore the normal tendon functionality for force transmission and body movements [4,14]. Given the anatomy and biomechanical function of the tendon, the development of a tendon biomimetic scaffold, which mimics the collagen parallel structure and the biomechanics of native ECM of tendon tissue, could be game-changing for tendon regeneration [15,16,17,18,19]. Scaffolds used in tendon TE are considered as temporarily ECM replacement whose aim is guiding the neo-formation and the deposition of ECM while parallelly degrading throughout the regeneration process [20]. These could be addressed through two complementary pathways including the activation of endogenous and/or exogenous cell regeneration mechanisms that could be driven by the functionalization of the scaffold with stem and/or progenitor cells [21]. Therefore, scaffolds are frequently utilized in combination with growth factors and stem cells whose aim is to support the tissue healing both mechanically and biologically. Engineering functional tendon scaffolds will thus require new approaches for the design of scaffolds with multi-scale resolution and biomimetic features in terms of biophysical and biological properties, as well as cellular regeneration to control cell spatial distribution and/or signaling molecules for modulating cellular fate. Only a highly integrated approach that can combine under a single system the multiple biophysical, biochemical, and biological cues controlling tenogenesis and inflammation will allow the fabrication of functional tendon biomimetic scaffolds that can be transplanted in vivo. Recent studies have demonstrated that modulating the intrinsic (fiber alignment and diameter size, pore size, surface modification, incorporation of bioactive molecules) and extrinsic (scaffold degradation, oxygen concentration and mechanical stimuli) features of the designed scaffolds [22] activates an inflammatory cascade at the injury site, which in turn might negatively or positively affect tendon regeneration [23,24].

In this context, immunoengineering has emerged as new discipline, which involves different strategies that enhance the interaction between the transplanted biomaterial-scaffold and the host tissue, hence modulating the immune response and hindering fibrosis occurrence at the injury site [25]. An ideal scaffold must change the tendon pro-inflammatory response and enhance its regeneration. Understanding the effect of scaffolds on modulating the immune response and promote tendon regeneration is fundamental, as it allows to fabricate “immunoinformed” scaffolds that incorporate specific design characteristics to actively modulate the host tissue immune response and are able to co-adjuvate the switch from a pro-inflammatory response towards a pro-regenerative one [26]. Indeed, when implanting a biomaterial scaffold, the immune system is the first responder to the foreign body [22]. The behavior of immune cells, that have large and diverse secretomes, can be tuned by the intrinsic and extrinsic features of the biomaterial scaffold [22,25]. Targeting these early responders to the scaffold can influence the local microenvironment and attract key contributors in the subsequent regeneration process, such as stem cells and vascularization [27,28]. This less explored feature in TE refers to applying immunoengineering biomaterial strategies, in which optimally designed and functionalized scaffolds allow to modulate host inflammatory response and stimulate tissue regeneration [27,29,30,31] or even potentiate stem cells’ immunomodulatory functions [32,33].

**Figure 1:**
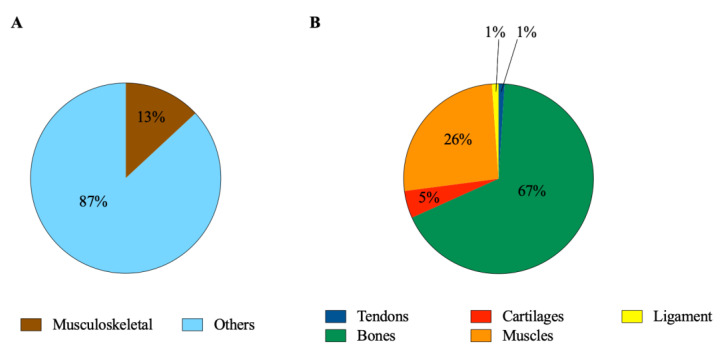
The comparative scientometric analysis of available publications on the Scopus database by using the terms “immunomodulation”, “immunomodulatory”, “immunoregenerative”, “immunoregeneration”, and “immunoengineering” reveals that: (**A**) only 13% of the total publications refer to the musculoskeletal tissues. (**B**) A deep analysis concerning the musculoskeletal tissues demonstrated that bones are the most studied tissue in this field (67%) followed by muscles (26%), cartilages (5%), and finally with the least publications number for tendons and ligaments with 1% each.

The relevance of the topic can be deduced by analyzing the Scopus database and performing a scientometric approach on the available literature using “immunomodulation”, “immunomodulatory”, “immunoregenerative”, “immunoregeneration”, and “immunoengineering” as keywords. The analysis revealed that 16,352 out of 124,997 (13%) belong to the musculoskeletal tissues (Figure 1A). A deep detailed database research allowed to determine that bones, muscles, and cartilages cover 67%, 26%, and 5%, respectively, whereas the remaining percentage (2%) belong to tendons and ligaments (Figure 1B).

**Figure 2:**
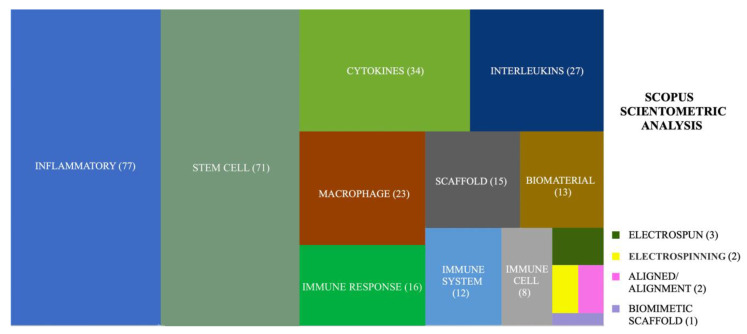
The scientometric analysis conducted on Scopus database (304 total articles) with the aim to assess the different research topics concerning the immunoregeneration of tendon discussed in this review. The legend indicates the different keywords used in the research whereas the number of total publications for each keyword is written inside the box.

A further in-depth analysis of manuscripts available on tendons and ligaments was used to identify the main topics to design the present review in which a total of 304 articles were found (Figure 2). In more detail, 197 articles (65%), belonging to the following keywords “inflammatory”, “cytokines”, “interleukins”, “macrophage”, “immune response”, “immune system”, and “immune cell” concern the immune response impact tendinopathy-mediated inflammatory reaction. Moreover, 71 articles (23%) consider the stem cell role in improving tendon healing and regeneration by hindering and modulating the inflammatory response in vivo. The remaining 36 articles (12%), which represent the keywords “scaffold”, “biomaterial”, “electrospinning”, “electrospun”, “aligned/alignment”, and “biomimetic scaffold” focused on scaffolds and, in particular, on electrospinning made materials by considering their effect in modulating the immune response in tendon ruptures. Hence, it is of great importance to put light on the importance of tendon-like scaffolds and controlling their characteristics in modulating the inflammatory response of the implanted scaffolds in tendon in vivo applications.

Starting from these premises, the present review is designed to give an overview about the scaffolds’ immunoengineering strategies for tendon TE applications, with focus on electrospun scaffolds, applied to modulate the immunomodulatory properties of the different immune and stem cells in vitro and in vivo. Moreover, the different molecular pathways regulating the scaffold mediated immunomodulation are detailed and discussed.

## 2. Biomimetic Scaffolds Applied for Tendon TE

When designing a scaffold for tendon TE applications, many characteristics need to be fulfilled, in particular concerning scaffold surface morphology and mechanical properties to mimic the native tendon tissue properties and fibrous structure. The tendon ECM, which constitutes around 80% of the tendon, contains predominantly collagen, which represents 60% to 85% of the dry mass, while the remaining approximate 20% represents the resident cells including tenocytes, tenoblasts, tenocyte progenitor/stem cells (TPSCs), and endothelial cells [34,35]. Collagen molecules within the tendon are arranged hierarchically, and intercalated with a less fibrous, highly hydrated matrix, traditionally referred to as the ground substance [36]. Collagen type I (COL1) is the most abundant collagen molecule and is responsible for the fibrous structure [37,38], followed by collagen type III (COL3) which is normally restricted to the endotenon and sheets. In case of spontaneous tendon healing, instead, COL3 is abundantly found representing the first collagen to be produced in high quantity [38,39]. In this phase, COL3 fibers are randomly arranged forming a sort of scaffold for the repairing site, accompanied with an increased cellularity. When the remodeling stage occurs during the tendon healing, there is a decrease of cellularity and matrix production. At this stage, COL3 is replaced by COL1 although with a favorable higher ratio of COL3. This condition induces a reduced strength of the repaired tissue. As a consequence, the tendon thickens and has a lower mechanical strength; thus, the tendon quality and its functional activity are inferior to that of a healthy tendon [38,39].

Healthy tendons are characterized by their elasticity, good flexibility, and high mechanical strength. Indeed, the materials must have appropriate mechanical properties, comparable to tissue target of the regeneration and sufficient to maintain the morphology of the biomaterial during tissue development [34,40].

The tendon biomimetic scaffold must be designed with teno-inductive characteristics to induce recruited progenitor cells or use undifferentiated stem cells for their differentiation towards the tenogenic lineage [16,17,40,41]. Moreover, the designed tendon-like scaffold should be teno-conductive to promote tendon growth and the surrounding tendon ingrowth, thus directing neo-tendon deposition [17,19,41,42,43]. The tendon-like scaffold to be implanted should be integrated into the surrounding host tendon. The teno-integration property depends on teno-induction and teno-conduction. It is referred to the direct anchorage of tendon within the damaged area without the formation of a scar fibrotic tissue [41].

A key important aspect to be considered when designing tendon-like scaffold is the immunomodulation [22]. Two concepts should be taken into consideration: immunotolerance and immunoinduction. The fabricated tendon-like scaffold should be immunotolerated, so that it should not induce an inflammatory response within the implanted site by reducing the activation of immune cells after transplantation. Additionally, the scaffold should be immune-inductive, thus designed to modulate a favorable immune response by regulating the intracellular and cell surface receptors presented on the host immune cells, such as toll-like receptors (TLRs), dendritic cells (DCs), antigen-specific T-cell receptor (TCR), and B-cell receptor (BCR), as well as by supporting immunomodulation, for example by shifting the pro-inflammatory M1 macrophages into anti-inflammatory M2 [24,31,44,45], which is discussed in detail in the next chapter.

Although different techniques have been used to fabricate scaffolds that aim to replace damaged tendons including sponges [43,44], freeze-drying [45,46,47,48], extrusion [49], and electrochemically aligned collagen [50,51,52], electrospinning is considered one of the most effective technique due to its versatility and applicability, as well as its ability to produce fibrous matrices that resemble native tendon architecture, with the possibility to control the fiber orientation and alignment [17,18,19,40,53,54,55,56,57].

The achieved fibrous structure should mimic the hierarchical morphology of the native tendon tissue characterized by collagen fascicles, fibers, and fibrils [58,59,60], confirming the well-known biomimicry characteristics of the electrospun fibers relevant to support cell adhesion, proliferation and differentiation [42,50,61,62,63,64,65].

The understanding of the relationship between scaffold properties and cell differentiation towards tenogenic lineage is relevant because the scaffold properties can be tailored by optimizing the electrospinning process parameters. The fiber orientation is a key point for the obtainment of a scaffold suitable for tendon TE and it is usually adjusted by an appropriate selection of the fiber collector type and configuration. Typical electrospun fiber diameters can range from 10 nm to 10 μm [66]. In tendon TE, the electrospun fibrous scaffolds should be in the micrometer range since microfibers mimic the physiological architecture of collagen fibers where tenocytes reside [18,62]. Indeed, it has been demonstrated that electrospun microfibers allow higher cell alignment and teno-differentiation accompanied with an improvement in the alignment of ECM, avoiding the formation of scar fibrotic tissue and promoting tissue healing [62,67]. Instead, electrospun nanofibers, which mimic collagen fibril size, have shown to stimulate the proliferative phase of tendon repair [62,67].

The electrospinning technique allows the fabrication of scaffolds, which possess different biomimetic tendon-like shapes adequate to be used for tendon TE applications [17,18,19,54,68,69,70,71,72]. Indeed, bundles and yarn scaffolds are other types of tendon-like scaffolds characterized as filament and twisted filaments of electrospun aligned fibers, respectively, which better mimic the hierarchical tendon architecture, as for example tendon fascicle crosslinked electrospun collagen nanofibers [56,57]. Multilayer scaffolds [73], stacked and braided scaffolds, represent scaffolds used in tendon TE obtained by assembling meshes of electrospun aligned fibers using crosslinking approaches to improve their mechanical properties [42,74,75].

## 3. Immune Response Induced by Scaffold Implantation

**Figure 3:**
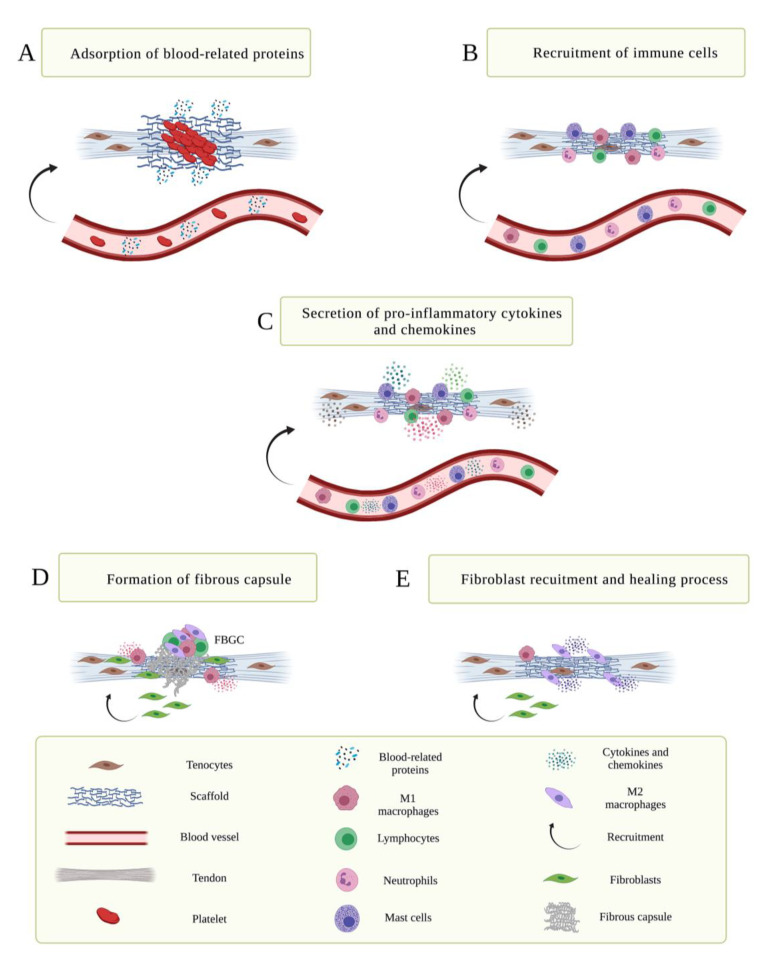
Interaction between scaffold and host tissue after implantation. The host starts a cascade of reaction called foreign body response (FBR). (**A**) Blood-related proteins and circulated platelets are recruited at the implantation site and are adsorbed on the surface of the scaffold allowing the activation of the coagulation process. (**B**) Immune cells are recruited at the implantation site and are accumulated between the scaffold and the surrounding tissue. (**C**) The immune cells start to secrete pro-inflammatory cytokines and promote inflammation. (**D)** The protracted presence of the scaffold and the persistent inflammation accompanied with the increased number of M1 pro-inflammatory cytokines leads to a continuous activation of tenocytes which secrete more collagen, which contributes to the formation of fibrous capsule and the rejection of the scaffold. (**E**) The switch of macrophages towards the anti-inflammatory/pro-regenerative phenotype M2 promotes tendon regeneration and facilitates the healing process.

The implantation of scaffold in vivo starts a cascade of reaction called foreign body response (FBR), evolved by the host tissue that usually lasts for 1 or 2 weeks (Figure 3). FBR can either determine the failure of the implanted scaffold or progress in tissue regeneration mainly induced by the shift from pro-inflammatory M1 macrophages to anti-inflammatory M2 macrophages and T helper cells (i.e., Th2) [32,38]. In this field, researchers are working on developing new strategies that aim at suppressing and harnessing the immune system to promote scaffold tolerance. Immunotolerance represents the functional unresponsiveness of the immune system towards cells and tissues [76]. Scaffolds should avoid the risk of inducing an aberrant inflammatory response when being implanted in vivo. A lack of an immunomodulatory response in terms of macrophage M2 and Th2 results in frustrated phagocytosis, which is detrimental to tissue repair [32].

In this process, innate and adaptive immune response are both in charge [32]. In detail, when a scaffold is implanted in a host tissue, blood-related proteins are adsorbed on the surface of implanted scaffold which activate the coagulation process allowing the formation of a temporary matrix characterized by an initial adsorption of albumin, substituted then by globulins that are in turn replaced by fibrinogen, fibronectin, factor XII and high molecular weight kininogens (Figure 3A) [77]. Fibrinogen accumulation represents a key role in FBR; its spontaneous adsorption appears to initiate the acute inflammatory response. Innate immune cells, including neutrophils, mast cells, and monocytes/macrophages are then recruited and accumulate in the region between the scaffold implants and the surrounding tissue (Figure 3B) [78]. The recruited cells start to secrete pro-inflammatory cytokines and chemokines, hence increasing the immune cell recruitment and promoting inflammation (Figure 3C) [32]. Afterwards, immune cells from the adaptive immune response including B cells, CD4+ and CD8+ T cells, natural killer (NK) cells, and innate lymphoid cells start to release cytokines and chemokines within the implanted site [32,79,80,81]. The occurrence of acute and chronic inflammation is followed by the formation of neovascularized connective tissue, called granulation tissue. The permanent implant could contribute to chronic inflammation documented by the presence of macrophages, lymphocytes, and foreign body giant cells (FBGC) (Figure 3D). These last, resulted from the fusion of adherent macrophages, are associated with the switch from pro-inflammatory M1 to pro-regenerative M2 phenotypes and from T helper 1 (Th1) cells to Th2, that could attempt to increase their regenerative functionality (Figure 3E) [24]. Both macrophage phenotypes are transient, which means that polarized macrophages will re-polarize to a different phenotype based on environmental needs. A previous work showed that the recruitment of pro-regenerative M2 macrophages promotes tendon regeneration [82]. Thus, in order to reach an optimal tissue regeneration, there is a need to tune the type and timing of the inflammatory components.

In this context, while much emphasis has been placed by researchers on varying electrospun scaffold properties to improve target cell adhesion, infiltration, integration and their teno-differentiative responses [34,40], it is crucial to focus the attention on the interaction between immune cells and a specific scaffold design [30,78]. Thus, understanding how to modulate scaffold microenvironmental cues to monitor the immune cell response, and in particular M2:M1 ratio, is crucial in the development of next-generation immunomodulated scaffolds able to positively promote tissue remodeling, incorporation, and regeneration [26,30]. The activation state of the inflammatory response can be further evaluated by analyzing the expression profile of released cytokines by the immune cells [33,83,84,85].

On the premise that tissue regeneration is intrinsically linked to the host immune response, the immunoregenerative process could represent a key to regenerative medicine strategies. Scaffolds must not only be passive supports for stem cells’ activity after implantation to boost tissue regeneration. Indeed, scaffolds can be designed in a variety of ways to modulate the inflammatory response as well as to influence stem cells’ activity. In a more advanced way, these biocompatible structures must be designed to modulate the immune cells’ response and avoid the procrastination of the inflammatory condition by inhibiting the secretion of inflammatory cytokines, formation of fibrous capsule and chronic inflammation [30]. Then, to reach this target, it is crucial to investigate the immune cell–biomaterial cross-interactions and the consequences on the host response. Tuning the degradation rate of the tendon-like scaffolds is another factor to take into account since it may affect tendon regeneration. While slow degradation rate is attributed to the formation of FBGCs accompanied with chronic inflammation response and the formation of fibrous tissue, scaffolds with faster degradation rate exhibit high cell infiltration within the construct, improving tendon regeneration [86].

## 4. Scaffold Immunoengineering Strategies for Tendon TE Applications

Immunoengineering has emerged recently as new discipline whose aim is to generate scaffolds with immune-modulatory properties to improve the interactions between the implanted scaffolds and the host immune systems to enhance the regenerative process. It aims at applying the principles and approaches of engineering by developing immunomodulated scaffolds that induce a favorable host immune response [25] and, consequently, tissue regeneration. This discipline fits well with tendon resolution, as in fact, any technological advancement in tendon TE has to take into consideration that tendon healing is associated to the blunting of the tissue inflammatory state [10,11]. Indeed, the immune system plays a central role in each tendinopathy stage and regulates the processes of tissue repair by immune cells and the secreted cytokines [10].

Although historically many researchers have worked on developing biologically inert implantable scaffolds, others still working on fabricating ideal bioactive scaffolds aim at not only preventing interaction between scaffolds and the immune system, but also at improving their biological effect in co-adjuvating a healing and regenerative process within the implantable site [25]. Two layers of specificity are the key elements for the safeness and effectiveness of the developed scaffold-based technology: the antigen specificity and the immunomodulatory specificity. While the first one is addressed to ensure that tolerance is limited to only the cells and the tissues to be protected, the second one aims at ensuring that, for example, regulatory T cells, without the cytotoxic ones, are stimulated for protection [25].

Thus, the immunoengineering biomaterial strategies have the aim, by using optimal scaffolds, to modulate host inflammatory response and consequently stimulate tissue regeneration [29,30] or even potentiate stem cells’ immunomodulatory function [17,31,32]. In fact, stem cells, including mesenchymal stem cells (MSCs) or amniotic-derived stem cells, as the amniotic epithelial stem cells (AECs), represent important cell sources that have been widely used in tendon applications, in which it has been demonstrated an improvement in the inflammatory response and an amelioration in the tissue regeneration due to their immunomodulatory properties [34,82,87]. The regenerative role of stem cells passes through several modulation mechanisms of the immune response through their paracrine functions which mediate their therapeutic potentials [82,87,88].

**Figure 4:**
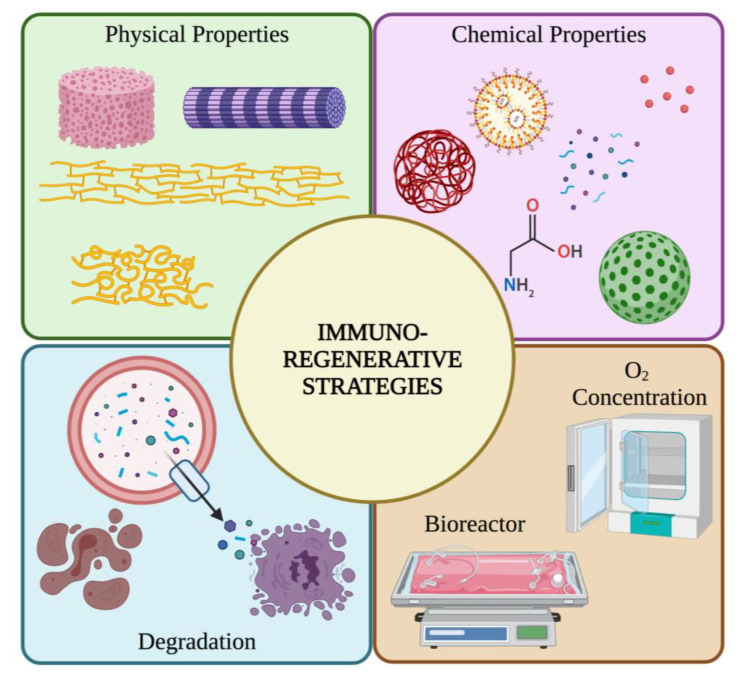
Immunoregenerative strategies applied in tendon TE to modulate the immune response of immune and stem cells.

The interplay of inherent electrospun scaffold properties with those arising from the interactions with the local environment, because of biomaterial interaction, is very complex. Different immunoinformed approaches have been studied and applied to produce immune-instructive niches made up of electrospun scaffolds including adjustment of: (a) the intrinsic properties of the scaffolds such as geometry, topography, porosity, pore size, substrate stiffness, and polymer and surface chemistry; (b) the temporal properties by modulating degradation rate of the scaffolds; and (c) the environment to which the scaffold is subjected including mechanical stimuli and oxygen concentration (Figure 4).

Based on these premises and considering the native tendon architecture, in the next paragraphs, only electrospun scaffolds with aligned topography and characteristics that mimic tendon ECM are considered and discussed in detail by exploring separately their effect either on immune (Section 4.1) or stem cells (Section 4.2).

### 4.1. Immuno-Induction of Scaffold on Immune Cells

**Figure 5:**
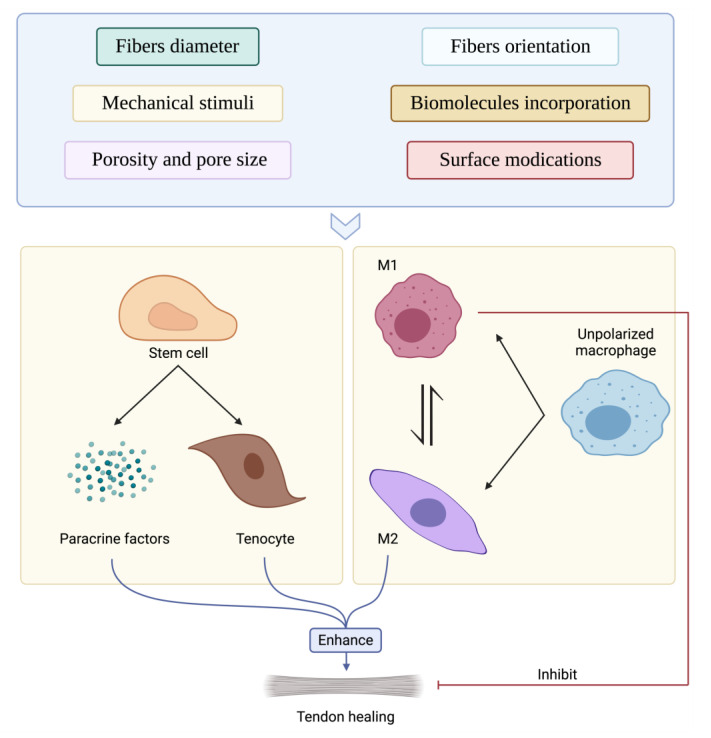
Effects of electrospun scaffolds-based immunoregenerative strategies on macrophage polarization and stem cell immunomodulation.

As described in the previous section, the inflammation and regeneration processes evolve different types of immune cells [89]. However, macrophages tend to be the most studied cell type due to their critical function in guiding tendon tissue regeneration and avoiding its fibrosis. In this section, the effect of intrinsic and extrinsic properties of electrospun scaffolds on immune cells is discussed with a particular attention on macrophages (Figure 5).

#### 4.1.1. Intrinsic Properties of the Scaffold on Immune Cells

##### Topography Effect on Immune Cells

Immunomodulation through topographical cues was recently discovered to be able to override the effects of surface chemistry in certain materials, especially in the first 6–48 h after initial contact. After implantation, topographical cues at the micro or nanoscale guide macrophage responses such as adhesion, spreading, activation, migration, and polarization. Indeed, macrophages, like many other cell types, can sense mechanical properties of their environment [30]. Murine macrophages tend to be unable to detect nano-topographical features smaller than 150 nm, while fibroblasts and endothelial cells can detect smaller topographies and show less spreading as feature size increases from 55 nm to 200 nm [30].

Surface topography properties such as fiber alignment and diameter have demonstrated a significant impact on the severity of inflammatory responses [24] and on the polarization of macrophages from inflammatory (M1) to anti-inflammatory/pro-regenerative (M2) and vice versa (Table 1). For example, Garg et al. cultured mouse bone marrow-derived macrophages (BMMΦs) on polydioxanone (PDO) scaffolds with different fiber diameter of 0.35, 2.20, and 2.80 μm, showing a positive correlation between increasing fiber diameters and expression of M2 markers [90]. Analogously, Wang et al. described an increase of RAW264.7 M2 macrophages phenotype when engineered on electrospun polycaprolactone (PCL) scaffolds with fiber diameter size of 5.59 μm rather than 0.69 μm, both in vitro and in vivo [91]. Instead, Saino et al. studied the effect of varying the topological properties of electrospun poly(L-lacticide) (PLLA) scaffolds in terms of fiber diameter and fiber alignment on the activation of macrophage RAW 264.7 and their secretion of pro-inflammatory cytokines and chemokines. In that study, four different types of fibrous PLLA scaffolds with different diameter sizes were identified as follows: aligned microfibers (1.60 ± 0.25 μm), aligned nanofibers (0.55 ± 0.16 μm), random microfibers (1.53 ± 0.32 μm), and random nanofibers (0.61 ± 0.18 μm), and PLLA film (0.2 mm thick flat surface) were engineered with macrophages RAW 264-7 for 24 h and 7 days culture. In comparison to films and microfibrous scaffolds, nanofibrous PLLA scaffolds reduced the inflammatory response. Furthermore, histological analysis revealed that the PLLA film showed a higher number of FBGCs than the micro and nanofibrous scaffolds. Thus, the findings of Saino et al. show that the diameter of electrospun PLLA fibers influences in vitro macrophage activation and pro-inflammatory molecule secretion [29].

Immune responses have been shown to also be influenced by fibers’ orientation. For example, Schoenenberger et al. investigated the different macrophages’ outcomes when seeded on aligned or randomly oriented electrospun PCL nanofiber substrates. In all the experiments, a disorganized biomaterial fiber topography was enough to facilitate a pro-inflammatory response in macrophages, tendon fibroblasts, and tendon tissue compared to aligned fibers [93]. Cao et al., compared the ability of electrospun PCL scaffolds with random and aligned fiber orientation on the adhesion of monocytes. Compared to randomly oriented fibers, aligned fibers showed a significant decrease in monocytes’ adhesion. The obtained results were also confirmed in vivo, showing that after 4 weeks, aligned fiber scaffolds were surrounded by a significantly thinner fibrous capsule. Moreover, the aligned fibers had more fibroblast infiltration, whereas the randomly oriented fibers accumulated cells on the surface [94]. In a similar way, as compared to surface-restricted geometries, the three-dimensional (3D) microstructure decreases inflammatory cytokine activity [30]. Another study on the orientation of scaffold was conducted by Jiang et al., who developed polymeric scaffolds by electrospinning PCL and then modifying the resulting fibers to give them different shapes: random or aligned. These scaffolds were then either left unmodified or expanded to macro-scale thicknesses of 3 or 10 mm, respectively (expanded in 1 M NaBH_4_ solution for 1 h at room temperature). Jiang et al., detected that expanded scaffolds promoted a regenerative answer and led to a thinner collagen fibrous capsule compared to unexpanded nanofiber scaffolds. Moreover, macrophages were able to penetrate scaffolds with randomly aligned fibers with extended thicknesses of 3 or 10 mm after subcutaneous implantation in rats. On the other hand, the scaffolds with aligned fibers that were expanded to 3 mm have shown to greatly support macrophage penetration with the fewest number of giant cells, which was attributed according to the authors to the gap distance between the aligned fibers [92].

The development of electrospun aligned fibers can be critical for the success of tendon tissue regeneration since they lead to a higher cell infiltration with a lower inflammatory response. The mechanisms through which this topography feature acts are discussed in the Section 4.

##### Influence of Porosity and Pore Size on Immune Cells

Porosity and pore size represent two critical scaffolds’ immunomodulatory factors that could affect not only the infiltration of biological molecules, including proteins or oxygen, but also the cellular behavior (Table 1). Size and frequency of the pores can be important cues for immunomodulation [100]. Porous implants are more easily vascularized and have less fibrous encapsulation than non-porous implants [30]. Garg et al., demonstrated that increasing pore size of PDO electrospun scaffolds (14.73 µm) directs macrophages polarization by boosting the M2 phenotype compared to other studied pore sizes (0.96 and 10.57 µm). This was suggested by the increased expression of Arginase I and down expression of M1 marker iNOS [90]. Therefore, this experiment revealed a link between increased pore size and a shift away from M1 macrophages toward M2 macrophages.

A main issue of the scaffold design is represented by the achievement of a suitable and high scaffold porosity, to maintain suitable mechanical properties for the target application. Tissue engineering constructs with increased porosity and an extended conformation can encourage pro-regenerative environments by altering macrophage function, as shown above. This improvement in scaffold structure, however, can have a negative impact on mechanical strength, and in the case of tissue-mimicking implants for structural components, the scaffold’s mechanical strength must match that of the native tissue [101]. Additionally, El Khatib et al. noticed that poly(lactic-co-glycolic acid) (PLGA) fiber diameter size is directly linked to the pore size of the fibers: by increasing the diameter, the pore size of the fibers increases as well [18]. This finding sounds very interesting since the study of El Khatib et al., also reported that doubling the fiber diameter (2.5 µm instead of 1.27 µm), and as a consequence the previously stated increase in the fibers’ pore size, resulted in worse mechanical features [18]. Therefore, this study is in accordance with Andorko and Jewell, and highlights that increased pore size can have a negative impact on scaffolds’ mechanical strength. The modulation of scaffolds’ porosity and pore size represents a good strategy for immunomodulatory purposes; however, it is necessary to find a compromise to obtain the best balance between the porosity of electrospun scaffolds and tendon-like mechanical properties.

##### Effect of Surface Modification and Biomaterial Chemistry on Immune Cells

For years, scientists have been working on changing the surface chemistry of implants to module the inflammatory response of immune cells. Indeed, surface chemistry/hydrophobicity has been long known to have a significant effect on the adsorption and denaturation of blood serum proteins, as well as on subsequent cell responses. In addition, surface roughness/smoothness influences wettability or hydrophilicity which in turn affects protein adsorption and cell adhesion [24]. Roughness is a propriety of the surface biomaterials that can be present on a microscale, microroughness, and that could enhance cell adhesion [100]. This propriety seems to be a stimulus factor for both M1 and M2 macrophages [100]. Several studies have shown that films with rough surfaces in the nanometer scale (150 nm to 4500 nm) can reduce fibroblast proliferation [102,103,104]. Smoother surfaces (roughness <200 nm) on the other hand have been shown to facilitate proliferation [98,99,101]. Furthermore, in vivo experiments have shown that when the surface roughness is greater than 500 µm, a thinner fibrous capsule forms [105]. The findings of these studies could be extended to electrospun nanofibers, but unlike films, these have a higher surface roughness.

In this field, friction plays a crucial role in tendon regeneration and adhesion formation. The friction between tissue and scaffold can be reduced by using a lubricant surface on the engineered biomaterial to reduce tissue adhesion (TA) formation. Failure to create friction-free motion results in TA, which in turn compromises the full tendon regeneration. Incorporating materials with low friction efficiency against the surrounding ECM, such as 5-fluorouracil and hyaluronic acid (HA), could lower protein absorption on the electrospun fibrous matrix (EFM) and minimize friction surface, leading to less tissue adhesion [85] (Table 1). For example, Deepthi et al. developed electrospun PLLA aligned nanofibers, mimicking the aligned tendon collagen fiber bundle, that were layered with a thin lubricating layer of chitosan (CTS) collagen and alginate hydrogel, simulating the glycosaminoglycans of sheath ECM for tendon regeneration. The study results showed a significantly reduced protein adsorption at the scaffold surface without affecting in vitro cell infiltration and attachment up to 7 days [97]. Protein adsorption experiments revealed that due to the lack of protein binding sites in alginate, protein adsorption onto alginate coated membranes was lower [85]. Moreover, Cheng et al., proposed a new method to improve the lubrication of electrospun PCL membranes by grafting 2-methacryloyloxyethyl phosphorylcholine on their surfaces [106]. Aside from the incorporation of different materials or surface chemical modification, appropriate physical structure design for EFMs could also reduce friction and promote tendon gliding [85]. Wang et al., for example, electrospun two layers of PLLA membranes and then used a shearing force to combine them into a single layer [99]. During the in vivo degradation process, such dual-layer scaffolds provided an artificial space to facilitate tendon gliding, which was beneficial for suppressing peritendinous adhesion and promoting tendon healing [85].

The biomaterial chemistry has a central role in the immune response; dependent on the different biomaterial nature, the inflammatory events could be modulated. Chemical properties of the material can affect macrophage function due to differences in the type and conformation of adsorbed proteins or charged regions on macrophage membranes. Currently, graphene is considered the most appealing material in nanotechnology [107]. Sub-cytotoxic concentrations of graphene family nanomaterials are able to modulate macrophages by stimulating the secretion of Th1/Th2 cytokines and chemokines most likely due to the TLR-dependent activation of the TLR-mediated and NF-κB signaling pathway [108]. Furthermore, it has been determined that a sub-cytotoxic dose of pristine graphene modulates macrophage morphology by modifying actin assembly and reducing their ability to adhere to the ECM [108]. Graphene oxide (GO), which represents the result of graphene oxidation to increase hydrophilicity and preparation of composites [109], has been shown to possess immunomodulatory properties, making them involved in the modulation of immune cells, neutrophils, macrophages, dendritic cells [110]. For example, GO sheets induce macrophage polarization to the M1 phenotype, enhancing the pro-inflammatory response [111]. Macrophage polarization towards the M1 lineage has also been observed with GO functionalized with amino groups, with an increase of secretion of CCL-5, IL-6, and IL-1β [112]. Furthermore, Mukherjee et al. determined that human neutrophils exposed to small (50–300 nm) and large (10–40 µm) sheets of GO produce neutrophil extracellular traps (NETs) that contribute in pathogen defense by immobilization and killing of bacteria [113]. These immunomodulatory properties together with the mechanical properties [114,115,116,117] of graphene and graphene-based materials have aroused a great interest in its usage in TE. It was only during this decade that GO was first incorporated into polymeric electrospun nanofibers [118]. Currently, several research teams are addressing their study on the evaluation of composites of this material with electrospun polymer scaffolds for tendon repair, for review [109]. Further studies are needed to investigate the employment other immunomodulatory biomaterials in the electrospinning technique. For example, the already mentioned CTS could represent a promising choice in TE scaffolds’ design [119]. However, CTS immunomodulatory effects have to be explored since its pro- or anti-inflammatory role depends on the context [119]. Moreover, there are only few reports about the electrospinning of pure CTS, and its usage in this field is limited due to its high viscosity [120].

#### 4.1.2. Effect of Scaffold Degradation on Immune Cells

The degradation rate of scaffolds can be influenced by the type of the polymer used. Moreover, the scaffold degradation rate should ideally be synchronized with the new healthy tissue formation, in fact the scaffold should provide sufficient mechanical and dimensional support to the new tissue, while it degrades [121]. The main synthetic polymers used for fabrication of electrospun scaffold for tendon TE applications belong to the aliphatic polyester family. These polymers are biodegradable from which one of the material’s basic component, lactic acid, is a cellular metabolite. These polyester-based scaffolds may provoke aseptic inflammation while degrading in vivo hindering, resulting in a complete regeneration, facilitating the formation of fibrotic tissue and inhibiting the development of COL1, the main component of tendon ECM [122,123,124]. It is hence thought to overcome the undesirable effect of released acidic by-product, during the degradation process of the scaffolds, to modify scaffold chemical properties by incorporating basic molecules able to neutralize acidic compounds, reduce inflammation, and enhance tissue regeneration [98] (Table 1). For example, Shen et al., fabricated shell-core structured fibers of CTS/PLGA with acid-neutralizing capability by coating the core surface of PLGA aligned fiber with a layer of the alkaline CTS using the coaxial electrospinning [98]. Indeed, even if PLGA represents an FDA-approved biomaterial and provides sufficient control of degradation [125,126], by this study, the pH decrease resulted from an 8-week PLGA degradation period that was impeded by the CTS layer maintaining the degradation medium pH at 6. Moreover, in vivo experiments conducted subcutaneously on mice for two and four weeks, showed a significant decrease in inflammatory cell recruitment and in the formation of FBGCs within the CTS/PLGA fibers. Therefore, this work reported the acid-neutralizing role of the chitosan-coating layer on lightening the inflammatory responses due to the PLGA by-products’ acidic degradation [98]. Jiang et al., conducted a study to understand which of these factors, namely the type of degradation by-products, material debris, and the acidic environment, are responsible for the subsequent immune reactions. The results showed that water-insoluble PLLA debris as well as water soluble PLLA/Polyethylene glycol (PEG) (1:1) debris are responsible for the regulation of the phagocyte activation and the subsequent tissue responses, as measured by reactive oxygen species production and inflammatory cell recruitment in both in vitro and in vivo models [127]. The findings that activated macrophages can phagocytose PLLA/PLGA particles [128] and that particle phagocytosis can induce the macrophages activation [129] backed up these observations.

#### 4.1.3. Effect of Environment Scaffold Subjection on Immune Cells

##### Mechanical and Electromagnetic Stimuli Influence on Immune Cells

Depending on the implantation site (i.e., presence of blood vessel, active joint loading or muscles) and tissue target of the regeneration, the scaffold may be subjected to dynamic or cyclical loading, resulting in cell dynamic or cyclical strain [130]. Mevoy et al. measured the impact of cyclical pressure on cytokine/chemokine production in cultured human macrophages, finding that all levels of cyclical pressure tested (17–138 kPa) resulted in an increase in pro-inflammatory cytokines (TNF-a, IL-6, IL-1β) compared to controls [131]. Researchers routinely expose electrospun scaffolds to mechanical stimuli in order to study the immunomodulatory consequence on the seeded cells (Table 1). Schoenenberger et al. studied the response of macrophages seeded on PCL nanofibers with aligned or random orientation, under conditions of mechanical load and unload (static). Macrophages were exposed to static (1% constant strain) or dynamic loading (7% cyclic strain at 1 Hz) for 8 h, followed by a 16 h quiescent period. Rather than the fibers’ alignment modulatory effect on macrophages, dynamic loading led to an upregulation of CCR7 (M1 marker), meaning the macrophage polarization through the pro-inflammatory phenotype [93]. Similarly, Bonito et al. assessed the effect of cyclic strains (0%, 8% and 14% strain at 0.8 Hz) on human peripheral blood mononuclear cells (hPBMCs) seeded on electrospun chain-extended (CE) ureido-pyrimidinone (UPy) modified PCL (CE-UPy-PCL) sheets with two different fiber diameters, 4 μm or 13 μm. They underlined a strain-dictated modulation of the inflammatory response (high strains addressed a pro-inflammatory condition) minimally affected by the different fiber diameters of the electrospun scaffolds [96]. In vitro and in turn in vivo experiments revealed that macrophages are mechanically responsive and change their lineages in response to mechanical stimuli. Mechanical stimuli in the form of tensile micromechanical strains, hydrostatic cyclic pressure, and compressive strains around the implant site further accelerate macrophage inflammatory cytokine synthesis [130], therefore the investigation of the macrophages’ response to those mechanical stimuli is fundamental for the design of an ideal scaffold [130].

Uniaxial and biaxial loading are the most common stimuli for tissues of the musculoskeletal apparatus (namely muscle, ligaments, and tendons). In detail, tendons and ligaments are subjected to uniaxial strain in the 5–16% range [130]. In this field, Ballotta et al. applied 7 and 12% cyclic uniaxial strains on hPBMCs seeded on a 25 mm by 5 mm 2D PCL-based tissue scaffold strip. Flexcell™ was used to apply mechanical strains at a frequency of 0.8 Hz for 7 days. They performed gene expression and immunohistochemistry analysis to study the expression of immune response markers in response to mechanical strain. Their findings indicated that a moderate amplitude of 7% mechanical strain facilitated hPBMCs’ polarization through the anti-inflammatory M2 phenotype and enhanced the expression of MCP-1, IL-6, IL-10, and MMP-9. In contrast, a 12% strain induced hPBMCs to acquire the M1 pro-inflammatory phenotype [95].

In a different way, another external trigger of the immune response has been found in the use of pulsed electromagnetic field (PEMF). These magnetic impulses can modulate inflammatory response in macrophages at low frequency. Vinhas et al. explored the potential modulatory effect of this technology in macrophage–human tendon cells (hTDCs) communication. They applied a magnetic stimulation regimen at 5 Hz, 4 mT, and 50% duty cycle for 1 h, using a magnetotherapy device. The PEMF resulted to influence a macrophage pro-regenerative phenotype (increased Arg-1, MRC-1, and Singlec-1 M2-like markers) and favors the synthesis of anti-inflammatory mediators (IL-6 and TNF α downregulation) [132].

##### How Oxygen Concentration Influences Immune Cells

A functionalized scaffold can provide in loco also changes in the local oxygen concentration. In detail, hypoxia can be used in cooperation with other factors to address and boost the tendon healing since tendons and ligaments are poorly vascularized tissues [133], with an oxygen consumption 7.5 times lower than skeletal muscles [134]. Assuming that skeletal muscle oxygenation is about 2–5% O_2_ (7.5 to 31 mmHg) [135,136], lower oxygen concentration appears to be crucial for a successful tendon recovery [34]. In addition, macrophages, in hypoxic conditions, accumulate and release pro-healing and pro-angiogenic factors such as VEGF and platelet-derived growth factor (PDGF), as well as enzymes such as cyclooxygenase 2 (COX-2). Hypoxia inducible factor (HIFs) 1 and 2, transcriptional factors, are thought to mediate macrophage responses to hypoxic environments. Although macrophages play a beneficial role in promoting vascularization of embedded scaffolds in a hypoxic wound setting, it should be noted that the hypoxic environment found in cancerous tissue actively recruits tumor-associated macrophages (TAMs) [26].

### 4.2. Immuno-Induction of Scaffold on Stem Cells

As described previously, stem cells, including MSCs [52,61,137,138,139] and amniotic-derived stem cells [17,18,19,140,141,142,143], have been widely used in tendon TE where they have shown teno-differentiative abilities allowing them to be a promising key element to be used in tendon regeneration [34]. Although they have the ability to differentiate towards the tenogenic lineage in vitro and in vivo, MSCs and amniotic-derived stem cells exhibited high immunomodulatory properties which in turn could enhance tendon regeneration [34,82,87,143,144,145,146]. Given the highly dynamic property of innate immune response and the immunomodulatory properties of stem cells, the combination of these last as well as their paracrine factors with tendon-like scaffold could represent an ideal microenvironment to module immune cells and the regeneration process within the tendon injury site. For this reason, new strategies must be developed by providing a suitable microenvironment for the tissue in regeneration, allowing an improved stem cell recruitment and their differentiation together with angiogenic reactions, avoiding any adverse inflammatory response [24].

The electrospun scaffolds can be designed not only to direct immune cell behavior and avoid scaffold rejection but can be also fabricated to tune and boost the immunomodulatory response of stem cells, which can in turn activate a new signaling cascade leading to the resolution of the damaged tendon tissue (Figure 5). Although many efforts have been made to assess the effect of physical and chemical properties of electrospun scaffolds on stem cell proliferation, migration and differentiation towards the tenogenic lineage [17,18,19,29,33,34,40,147] and their effects on regulating the immunomodulatory properties of stem cells must still be studied in depth. While researchers have focused their attention on the effect of intrinsic and extrinsic properties of electrospun scaffolds on affecting the cellular behavior of immune cells, few studies have evaluated the immuno-induction potential of this type of scaffold on stem cells by assessing mainly fiber alignment and diameter size as well as their exposition to mechanical stimuli.

#### 4.2.1. Intrinsic Properties of the Scaffold on Stem Cells

##### Topography Effect on Stem Cells: Fiber Alignment and Diameter Size

Stem cells belonging to bone marrow (BM) and adipose-derived (AD) MSCs together with AECs have been used to assess the effect of scaffold fiber alignment and diameter size on tuning their innate immunomodulatory properties [16,17,32,84,85]. The conducted studies revealed aligned topography rather than oriented fibers together with small fiber diameter size in the micrometer range of the electrospun scaffolds influenced the immunomodulatory properties of stem cells by regulating the gene and protein expression of anti-inflammatory cytokines compared to the pro-inflammatory ones, and by activating the mechanotransduction pathways during tendon regeneration (Table 2) [18,33,83,84,85].

Su et al., fabricated three electrospun PLC scaffolds with randomly oriented (R), aligned (A) and mesh-like (M) electrospun fibers (EFs) and they assessed their effect on the paracrine function of ADMSCs [83]. The obtained results showed that oriented AEFs and MEFs enhanced the expression of PGE_2_, iNOS, and VEGF within ADMSCs compared to those engineered within the REFs [83]. Moreover, the conditioned media (CM) obtained from the culture of ADMSC-MEFs and ADMSC-AEFs exhibited potent anti-inflammatory responses, characterized by elevated expression of IL-10 when cultivated with macrophages cells compared to REFs [83]. Similarly, in a study conducted by Wan et al., they demonstrated that ADMSCs engineered within PLLA electrospun scaffold with aligned fibers exhibited higher expression of immunomodulatory factors including PGE_2_, COX-2, TGF-β, macrophage colony stimulating factor (M-CSF) and TSG-6 [84]. TSG-6 acts as an inhibitor of the TLR 2-nuclear factor-B (NF-B) signaling in resident macrophages, reducing the typical activation of these cells into the phenotype M1 [148]. It also prevents neutrophil infiltration by interfering with the interaction of cell-surface glycosaminoglycans with a variety of chemokines from the CC and CXC subfamilies [149]. Regarding COX-2, it is a necessary enzyme to produce PGE_2_, a potent immunosuppressive mediator, which can modulate the phenotype of macrophages from M1 to M2 [150].

Recently, it has been demonstrated that also fiber size of electrospun scaffolds with highly aligned fibers affect the immunomodulatory profile of stem cells. For instance, El Khatib et al. demonstrated that changing diameter size of aligned electrospun PLGA scaffolds alters the interleukin profile of AECs [18]. The obtained results revealed that AECs seeded on electrospun PLGA scaffolds with small fiber diameter size (1.27 µm) expressed high levels of pro-regenerative, anti-inflammatory cytokines (IL-10) with favorable IL-12/IL-10 ratio compared to larger fiber size (2.50 µm), which induced on AECs’ high expression of pro-inflammatory cytokines (IL-12 and IL-6) [18]. The importance of lowering IL-12/IL-10 ratio appears to influence positively the inflammatory response of AECs by supporting their transition from an inflammatory to reparative phase, demonstrating a macrophage skewing towards the M2 pro-regenerative phenotype, as previously demonstrated by Manuelpillai et al. [151] in hAECs xeno-transplanted liver, and Mauro et al. [82] and Barboni et al. [140] observations in oAECs allo-transplanted and hAECs xeno-transplanted tendons, respectively.

#### 4.2.2. Effect of Environment Scaffold Subjection on Stem Cells

##### Mechanical Stimuli Influence on the Immunomodulation of Stem Cells

The remodeling of tendon ECM by the activity of MMP and TIMPs can be improved by applying mechanical stimuli. MMP and TIMPs are key elements for tendon tissue repair and maintaining their activities in balance is indispensable for healthy tendon homeostasis [152]. Tomás et al., fabricated electrospun PCL yarns incorporated with DT-nanoparticles (DT-NPs), engineered with human ADMSCs (hADMSCs) and cultivated under static and magnetic stimulation culture (Table 2) [33]. The obtained results showed that engineered hADMSCs-PCL-DT-NPs yarns, cultivated under magneto-mechanical stimulation, exhibited high expression levels of MMPs including MMP-1, MMP-2, and MMP-3 together with TIMPs when compared to engineered cells cultivated under static condition. Moreover, the analysis of pro- (IL-6 and COX-2) and anti- (IL-4 and IL-10) inflammatory cytokines showed that hADMSCs within PCL-DT-NPs yarns upregulated the anti-inflammatory cytokines’ gene expressions while downregulating those of pro-inflammatory ones, with a notable boosted expression of IL-4 and IL-10 under magneto-mechanical stimuli [33].

### 4.3. Biological Strategies to Enhance the Immunoregenerative Potential of the Scaffolds

Synthetic and natural biomolecules could represent an advanced strategy to modulate the host response of the damaged tissue due to the ability to tune their delivery timing at specific phases of inflammation and repair in vivo (Table 3). For instance, local releases may be used to create a gradient of a specific compound necessary to recruit monocytes and macrophages at a specific time within the injury site [30]. To this end, biological strategies have been developed with the aim to directly deliver bioactive molecules, chemokines, and nanoparticles, or incorporate them within the electrospun scaffolds providing a feasible microenvironment able to boost the immunomodulatory properties of transplanted or recruited cells within the injury or implantation site. The functionalization of the scaffolds with bioactive molecules can be considered as an intrinsic feature since it might be performed during or immediately after scaffold preparation.

Significant efforts have been also made to modify the synthetic biomaterial scaffolds with biological properties such as cytokines or anti-inflammatory drugs for delivering at the injury site or in a surface coated form [100].

Although these combinational strategies have been widely used for different TE applications, only a few studies have addressed the use of scaffolds with aligned fibers which can simulate the tendon-like structure of the ECM for immunoengineering [154,158]. One of these investigations functionalized the electrospun aligned fibers’ scaffold with 25-hydroxyvitamin D3 (25(OH)D_3_) [154]. This molecule has aroused great interest in the design of medical scaffolds that could modulate the immune response [167,168]. In vitro studies highlighted the role of vitamin D3 in the reduction of pro-inflammatory cytokines’ expression together with the increased production of anti-inflammatory ones [167,168,169]. Chen et al., evaluated the host responses using 25(OH)D_3_ eluting radially aligned electrospun PCL nanofiber scaffolds subcutaneously implanted in humanized mice [154]. The results showed that rather than significantly reducing the production of pro-inflammatory cytokines (TNF-a, IL-6), the production of anti-inflammatory cytokines (IL-4, IL-10) was boosted [154]. Indeed, in vitro and in vivo studies suggest that 25(OH)D_3_ exhibited a suppressive role on the expression of pro-inflammatory cytokines while raising that of anti-inflammatory cytokines [167,168,169]. For instance, Zhang et al. showed how 25(OH)D_3_ inhibited the production of the pro-inflammatory cytokines IL-6 and TNF-α from monocytes/macrophages, by upregulating mitogen-activated protein kinases (MAPKs) phosphatase-1 expression and suppressing p38 activation [169]. Another interesting molecule, able to be part of the immune response modulation, is melatonin. This hormone is largely secreted by the pineal gland, and has numerous biofunctions such as anti-inflammatory, antioxidation and immunomodulation properties [170]. Recently, melatonin has shown to inhibit the expression of pro-inflammatory markers in macrophages [171]. In order to obtain more information about this type of modulation, Song et al. cultured human bone marrow mesenchymal stem cells (hBMSCs) with melatonin-loaded aligned PCL electrospun fibrous scaffolds and tested them in vivo in a rat acute rotator cuff tear model. During the early healing phase, melatonin-PCL electrospun scaffolds could significantly inhibit M1 macrophage (CD68-positive cell) accumulation at the tendon-to-bone interface and improved the collagen fiber organization of the ECM [158].

Although most of the studies describing the incorporation of bioactive molecules were performed on randomly oriented fibers [85,153,155,157,161,165,171,172], which do not mimic tendon structure, their description can be of great interest since they could represent novel immunoinformed strategies to develop functionalized tendon biomimetic scaffolds aimed at boosting scaffold’s efficacy and its regenerative potential [85,153,155,157,161,165,171,172]. Within these investigations, a wide range of biomolecules used can be found. The release of factors (e.g., IL-4, IL-10, steroids) overwhelming the native signaling and directing macrophage polarization is a well-known and targeted methodology to control the immune response in an immunomodulated regenerative biomaterial [173,174]. In particular, the encapsulation of growth factors, gene delivery vectors, or small molecule drugs (e.g., steroids) in triggered release platform is the most used technique to achieve the above mentioned release of factors [175].

McWhorter et al. found that the morphological elongation of macrophages combined with M2 inducing cytokines (IL-4, IL-13) increased M2 polarization, meaning that biophysical cues directly presented by biomaterials may be used to complement the effects of factors already present in the native environment, in addition to directing polarization [176]. The biological response can be modulated to optimize repair by reducing the inflammatory response engineering material properties and biomolecule delivery [30]. To condition local macrophages to a specific phenotype, polarizing cytokines can also be released. For example, Qian et al., incorporated the anti-inflammatory cytokine IL-4 within silk fibroin-functionalized electrospun PCL with randomly oriented nanofibers using the layer-by-layer assembly technique that led in vitro to M2 macrophage polarization characterized by the detection of CD206 and Arginase I markers in a murine subcutaneous model [153]. Moreover, Bonito et al., developed an electrospun CE-UPy-PCL scaffold with randomly oriented fibers, functionalized with a UPy-modified heparin binding peptide (UPy-HBP) to immobilize IL-4 through the heparin binding domain. Therefore, after cultivating human PBMCs on the IL-4 functionalized scaffolds, the macrophages demonstrated to support an anti-inflammatory environment characterized by IL-10 upregulation at day 3, IL-6 downregulation, and TGF-β1 and MMP-9 overexpression at both day 3 and 7 after in vitro culture [172]. A further factor that overwhelms native signaling is the pro-inflammatory cytokine IFN-γ, which has been shown to induce the MSCs to secrete factors such as COX-2, PGE_2_, and indoleamine 2,3-dioxygenease (IDO) [177]. In the study carried out by Kim et al., hBMSCs were cultured on electrospun silk fibroin scaffolds (SFN) or PLGA nanofiber scaffolds, both of them randomly oriented, and treated with human IFN-γ [161]. In both situations, IFN-γ significantly increased the transcription levels of the immunomodulatory cytokines COX-2 and IDO, as well as the secretion of IL-10 compared with the control without scaffold. Instead, on the opposite side, it suppressed the secretion of TNF-α by the splenocytes [161].

Another study was carried out in vitro by Wang et al., in which they grafted basic fibroblast growth factor (bFGF) on the surface of electrospun PLLA scaffold with randomly oriented fibers to improve its hydrophilicity and assess its immunomodulatory potential [160]. A hydrophilic polypeptide, bFGF can stimulate angiogenesis, speed wound healing and tissue repair, enhance tissue regeneration and boost collagen production [178]. The creation of a constant bFGF release from the fabricated scaffold increased the anti-inflammatory-related cytokine TGF-β1 within human vaginal fibroblasts while decreased the concentration of TNF-α [160].

Traditional medicine has used olive tree products as botanical medications and food additives [179]. Olive leaf extract (OLE) has been studied for a variety of uses, including an anti-inflammatory drug, and it is well-known for being a good source of antioxidants, bioactive chemicals, and even polyphenols [179]. De La Ossa et al., investigated with randomly oriented electrospun polyhydroxyalkanoate (PHA) scaffolds functionalized with OLE, taking into consideration its polyphenols, more concretely oleuropein, as well as luteolin-7-O-glucoside and aspigenin-7-O-glucoside in lower concentrations. These scaffolds were cultured in vitro with human dermal keratinocytes (HaCaT cells) for a period of 24 h. When released by PHA fiber meshes, OLE has been shown to have diverse immunomodulatory effects in vitro, switching from pro-inflammatory to anti-inflammatory environments by downregulating IL-1, IL-6, IL-8 and TNF-α, and even stimulating defensin in the case of polyhydroxybutyrate (PHB)/poly(hydroxyoctanoate-co-hydroxydecanoate) (PHB/PHOHD) scaffolds [162].

A different approach for a scaffold functionalization was performed by Xi et al., where randomly oriented microsol electrospun fiber scaffolds (MSaP) were engrafted with IL-4 plasmid-loaded liposomes (aL/p) in order to assess in vitro their immuno-inductive properties. The functionalized scaffolds were cultured through a trans-well system with BMMΦs for a period of 7 days. The cells cultured on MSaP-aL/p groups exhibited a progressively decreased expression of pro-inflammatory genes IL-1 and TNF-α over time. Instead, the gene expression of anti-inflammatory gene IL-10 and the transforming growth factor (TGF)-β increased within cells cultured on MSaP-aL/p compared with the control [157].

Nonsteroidal anti-inflammatory drugs (NSAIDs), such as ibuprofen and celecoxib, can reduce TA formation by reducing inflammation and in turn by regulating the healing microenvironment, as showed by Zhang et al. who reported the loading of NSAIDs into poly(lactic acid-co-ethylene glycol-co-lactic acid) (PELA) EFMs [85]. Furthermore, ibuprofen-loaded electrospun PLA EFMs limited in vivo macrophage adhesion, proliferation, and infiltration by inhibiting TNF-α expression and collagen type III deposition, and reduced the inflammation and granuloma formation in the area surrounding the tendon [155].

Exosomes, known as membrane-enclosed extracellular vesicles (EVs) that carry proteins and nucleic acids, might represent another type of delivery system of bioactive molecules [180]. Su et al. developed biofunctional scaffolds by fixing mesenchymal stromal exosomes (Exo) to electrospun randomly oriented fibrous polyester materials (PEF) [156]. The fabricated scaffolds were examined in skin injury models of sham mice to understand the immunomodulatory effects of mesenchymal stromal exosomes (Exo) in vivo [156]. MSC-associated immuno-moderation activity supports the M2-like phenotype of macrophages, population of T_reg_ and immunological Th2 responses [181]. In fact, Su et al., demonstrated that Exo-PEF increased the number of immunomodulatory CD206+ M2 macrophages by a factor of one. On day 7, Exo-PEF also increased the number of T_reg_ cells by more than 3-fold compared to untreated wounds, as well as the proportion of T_reg_ cells secreting the anti-inflammatory cytokine IL-10. Furthermore, compared with untreated mice, Exo-PEF promoted the secretion of IL-4, IL-10, and IL-13 and decreased the pro-inflammatory chemokines TNF-α and IFN-γ [156]. Another example of exosome involvement was shown by Chamberlain et al., which generated M2-like macrophages, avoiding the usage of MSCs directly, by using exosomes isolated from MSCs and creating exosome-educated macrophages (EEMs) [182,183]. For example, the researchers next compared the effects of EEMs on mouse Achilles’ tendon rupture to normal tendon healing, MSCs, and EVs. Exogenous delivery of EEMs directly into the wound enhanced tendon mechanical characteristics, reduced inflammation, and accelerated angiogenesis, according to Chamberlain et al., whereas treatment with MSC-derived EVs alone was less beneficial while reducing the M1/M2 ratio [182]. Moreover, Shen et al. assessed the effect of adipose stem cells’ (ASCs) EVs on early tendon healing using a mouse Achilles tendon injury and repair model [184]. Tendon healing was evaluated in nuclear factor-kB (NF-kB) luciferase reporter mice, up to 7 days after surgery. Following tendon repair, NF-kB activity increased by more than threefold [184,185,186], the reaction was effectively reduced by using EVs from primed but not naïve ASCs. Furthermore, in repaired tendons, the pro-inflammatory genes Il-1b and IFNG were both markedly elevated, whereas primed, but not naive ASC EVs reduced the response [184]. Several studies have demonstrated the effect of immunomodulatory properties of MSCs on reducing the inflammasome in macrophages by modulating macrophage polarization [159,187,188,189,190,191]. However, only the study conducted by Dong et al. implied the functionalization of randomly oriented electrospun PCL/silk fibroin scaffolds with decellularized ECM derived from hBMSCs (PCL/SF-ECM) (Table 3) [159]. In this study, the role of bioactive molecules contained within the PCL/SF-ECM scaffold was assessed on modulating macrophages’ polarization for tendon applications. The fabricated scaffold enhanced M2 macrophage polarization and reduced the expression of multiple cytokines (IL -1β, IL-6, CXCL11, IL-10, IL-1R2 and TGF-β1) in vitro [159]. TGF-β1 is a profibrotic factor that stimulates fibroblast recruitment and in turn collagen secretion through the Smad3 pathway [192,193]. Indeed, probably because of scaffold inhibitory effect on TGF-β1 expression, the results of rat subcutaneous implantation showed a lower FBR, a thinner fibrotic capsule formation, and a higher M2 macrophage phenotype polarization [159]. Furthermore, Aktas et al., used a rat Achilles segmental defect model to assess the in vivo healing benefits of TNF-α-primed MSCs [194]. Rat Achilles tendons were damaged and then repaired using a PLGA scaffold alone, an MSC-seeded PLGA scaffold, or a TNF-α-primed MSC-seeded PLGA scaffold. Samples were analyzed two and four weeks after the injury. MSCs boosted the production of IL-10 while decreasing the inflammatory factor IL-1a while primed MSCs decreased the production of IL-12 and the number of M1 macrophages simultaneously increasing the percentage of M2 macrophages and IL-4 synthesis. When TNF-α-primed MSCs were delivered via 3D PLGA scaffold, macrophage polarization and cytokine production were regulated, enhancing the more regenerative MSC-induced healing response [194].

Apart from the studies regarding the effects of the bioactive molecules on improving the teno-inductive potential of electrospun scaffolds, other investigations concerning the use of different kinds of biocompounds to improve the modulatory effects of the immune system in tenocytes and stem cells were performed [163,164,165,166]. As previously mentioned, some pro-inflammatory molecules such as IFN-γ induce the secretion of immunomodulatory proteins, such as COX-2, PGE_2_ or IDO [177] in the MSCs. An experiment in which BM-MSCs exposed to IFN-γ and TNF-α was performed. At low concentrations of these cytokines, there was an upregulation of the immunomodulatory genes IDO, iNOS, IL-6, COX-2 and VCAM-1. There was also an increase in the expression of MHC-II and CD40 [163]. Another molecule with promising therapeutic uses is platelet-rich plasma (PRP), which is renowned for influencing the early healing response by secreting a variety of signaling cytokines that regulate inflammation and angiogenesis at the same time, as well as cell migration and proliferation [195]. In a research study conducted by Andia et al., the immunomodulatory effects of PRP were assessed in vitro on tenocytes exposed with or without IL-1β to induce an inflamed phenotype [164]. The treatment with PRP reduced the expression of pro-inflammatory interleukins including IL-6, IL-6R, and IL-8 in the IL-1β exposed cells. The secretion of IL-6, IL-8, and monocyte chemoattractant protein-1 was also reduced after PRP treatment, whereas VEGF increased 2-fold. In tendinopathic cells, regulated upon activation, normal T cell expressed and secreted (RANTES), representative of C–C chemokines, grew 10-fold and hepatocyte growth factor (HGF) increased 21-fold, while in normal cells increased 2.3-fold [164]. Another bioactive molecule was studied by Zarychta-Wiśniewska et al., which treated human ASCs (hASCs) with Bone morphogenic protein 12 (BMP-12) in order to determine in vitro its tenogenic modulation, as well as its induced immunomodulatory on hACSs [166]. The BMP-12 belongs to a protein group that can induce ectopic formation of tendon-like structures and improve healing parameters in injured tendons [196]. The results showed that BMP-12 induced in vitro tenogenic differentiation of hASCs, and stimulated VEGF, MMP1, MMP8 and IL6 secretion within the cells substantially. Instead, no effect was detected in the transcription levels of EGF, IL-10, TGF-β and MMP-13 [166]. In a different scenery, Zhang et al. performed an experiment in which tendon fibroblasts were cultured in a conditioned medium from tendon stem cells enriched with a gradually increased concentration of HGF [165]. In these circumstances, there was an upregulation of MMP-2 and MMP-9, α-SMA, TIMP-1 and VEGF in the ECM, as well as the anti-inflammatory cytokines such as IL-10. On the other hand, a downregulation of the pro-inflammatory cytokines such as IL-6 was observed. These results were verified both in vitro and in vivo, with a more notable effect with the higher concentrations of HGF [165].

Another method to functionalize scaffolds with bioactive molecules is implicated by their encapsulation or loading NPs to control their delivery and release to modulate the inflammatory response that might occur at the injury site [197]. NPs have been widely used in different applications including the production of sensors, materials construction, as well as in the biomedical field for different applications such as drugs delivery, vaccine adjuvants or catalysts to boost chemical reactions [198]. The use of NPs in regenerative medicine has been increasing over the years, thanks to the improvements of the synthesis techniques, as well as the new FDA approved materials used [199]. In some cases, NPs themselves can exhibit immunomodulatory potential. A study concerning this topic in a tendon-like environment was performed by Vinhas et al., where they developed magnetically assisted cell sheets (magCSs) by using hTDCs with magnetic NPs (MNPs). MagCSs were in vitro subjected to IL-1β to induce an inflammatory-like environment and then were exposed to a PEMF. The results showed that under these conditions, the levels of pro-inflammatory factors IL-8, IL-1β, TNF-α, and IL-6 decreased, whereas the transcription levels of anti-inflammatory factors IL-10 and IL-4 increased. Thus, PEMF appeared to successfully restore anti-inflammatory factor levels in inflammatory conditions, considered crucial for the tendon healing process [200]. A similar study conducted by the same research group developed magnetic membranes of a polymeric blend of PCL and starch (S) with iron oxide nanoparticles (magSPCL), which were cultured in vitro with hTDCs and exposed to PEMF [201]. The levels of the pro-inflammatory molecules TNF-α, IL-6, IL-8 and COX-2 decreased, while the expression of anti-inflammatory cytokines (IL-4 and IL-10) increased. The results indicate that magSPCL exposed to PEMF can successfully suppress the expression of NF-κB, a transcription factor that regulates and coordinates the expression of various pro-inflammatory genes and mediators, including cytokines, chemokines, adhesion molecules, immunoreceptors and growth factors [202]. Indeed, these conditions were used to assess the effect of magnetic membranes on macrophages. These last showed a more elongated shape, associated with a pro-healing phenotype, when cultured under the above-described conditions. Moreover, macrophage behavior was confirmed by the increased expression of the surface markers CD16^+^, CD169^+^ and CD206^+^, indicating their polarization towards the M2 phenotype [201].

In addition to the immunomodulatory effects of nanoparticles under specific conditions, other studies have focused on their role in the delivery control of bioactive molecules and chemical compounds. In a study conducted by Kang et al., porous PLGA microspheres (PMSs) were synthetized with the immobilization of heparin-dopamine (Hep-DOPA), and platelet-derived growth factor (PDGF) (PDGF/Hep-PMSs) to in vitro examine the inflammatory responses on LPS-stimulated rabbit tenocytes [203]. The results showed a high degree of immunomodulation characterized by suppressing the mRNA levels of six pro-inflammatory cytokines MMP-3, MMP-13, COX-2, IL-6, TNF-α and A Disintegrin and Metalloproteinase with ThromboSpondin motif (ADAMTS-5), while increasing the mRNA levels of anti-inflammatory cytokines IL-4, IL-10, and IL-13 [203]. A comparable study concerning the same type of NPs was performed by Jeong et al., where they developed simvastatin-loaded porous PLGA microspheres (SIM/PMSs) on (LPS)-treated tenocytes [204]. Simvastatin belongs to the drug class of statins (used to lower blood cholesterol), and exhibits anti-inflammatory properties accompanied with side effects when administrated at high doses [205]. The research was conducted to assess (LPS)-treated tenocytes’ immune response at different concentrations of SIM/PMSs and showed that the outcome of the microspheres had a dose-dependent decrease in the mRNA levels of MMP-3, COX-2, IL-6, and TNF-α, with stronger effect at 5 nM compared to 1 nM. Additionally, in an in vivo model of RC tendinopathy there was a slight increase in the mRNA levels of the anti-inflammatory cytokines IL-4, IL-10, and IL-13 [204]. In a further study using the PLGA material, Jong Choi et al. fabricated lactoferrin-immobilized, heparin-anchored, PLGA NPs (LF/Hep-PLGA NPs) [206]. These bioactive molecules were anchored together, as well as separately, in order to evaluate their effects on IL-1β-treated tenocytes in vitro, and in vivo through a rat model of Achilles tendinitis [206]. Independent from the study conditions (in vitro and in vivo), the nanoparticles containing lactoferrin (LF-PLGA NPs) and LF/Hep-PLGA NPs managed to decrease the mRNA levels of the pro-inflammatory factors COX-2, IL-1β, MMP-3, MMP-13, IL-6, and TNF-α, while increased the levels of the anti-inflammatory cytokines IL-4 and IL-10 [206]. Another commonly used anti-inflammatory drug is Diclophenac Diethylammonium. By fixing this medicine into gold nanoparticles (GNPs), rats with a tendinous injury model were treated using a pulse therapeutic ultrasound technique, in which the drug was transported transcutaneously [207]. A significant decrease in the pro-inflammatory cytokines IL-1β and TNF-α occurred in tendons with the phonophoresis + diclophenac + GNPs treatment, and these nanoparticles showed to be effective in transporting molecules to active inflammation sites [207].

Apart from the treatments with nanoparticles alone, a study conducted by Ciardulli et al. addressed the use of NPs in scaffolds to study the immunomodulatory properties of this combination. They developed a hyaluronate elastic band merged with a fibrin hydrogel scaffold (HY-FIB) containing hBMSCs and PLGA nano-carriers (PLGA-NCs) loaded with human Growth Differentiation Factor 5 (hGDF-5) which has been shown to induce the expression of genes linked to the neo-tendon phenotype [139,208]. This construct was studied either in static or dynamic conditions in a 3D in vitro model to understand under which settings exist the better tendon phenotype, as well as a pro-healing cytokine profile. The results demonstrated that in dynamic conditions, pro-inflammatory cytokines IL-6, TNF, IL-12A, and IL-1β displayed a less pronounced upregulation, while anti-inflammatory TGF-β1 and IL-10 showed an increase by day 11 [139].

Altogether, bearing in mind the immunomodulatory possibilities offered by nanoparticles/microspheres and electrospun scaffolds, more preliminary experiments using these approaches in combination could be performed, hence allowing the obtention of a potential synergic effect in modulating the immune system together with a different cell compartment within a tendon-like environment. Under these terms, Tomás et al. functionalized aligned electrospun fiber threads of PCL matrix filled with iron oxide magnetic nanoparticles (MNPs) attached to cellulose nanocrystals (MNP-CNCs) [33]. This 3D scaffold was seeded with hASCs and activated with a magneto-mechanical stimulation. After 11 days, cells cultivated on PCL/DT-NP5 under both static and magnetic conditions presented increased expression of tendon-related markers. Furthermore, similar to the tenogenic differentiation, the magnetic stimulation showed a higher immunomodulatory potential, increasing the expression of anti-inflammatory cytokines IL-4 and IL-10, while downregulating the pro-inflammatory cytokines IL-6 and COX-2 [33].

Considering the role of bioactive molecules together with the influence of the scaffolds’ intrinsic and extrinsic characteristics in immunomodulating the response of different cell types both in vitro and in vivo, it is a promising idea for future experiments to study the synergic effect that could have the electrospun scaffolds with aligned fibers engrafted with these kinds of bioactive molecules on modulating the immune system response in tendinopathies. Thus, the core concept in designing scaffolds, in combination with engineered bioactive molecules and/or stem cells, resides in the modulation of the interaction between the transplanted biomaterial-scaffold and the host tissue. This in turn allows a pro-regenerative immune response, hence hindering fibrosis occurrence at the injury site and guiding tendon regeneration.

## 5. Insights in the Molecular Pathways Regulating the Scaffold’s Mediated Immunomodulation

**Figure 6:**
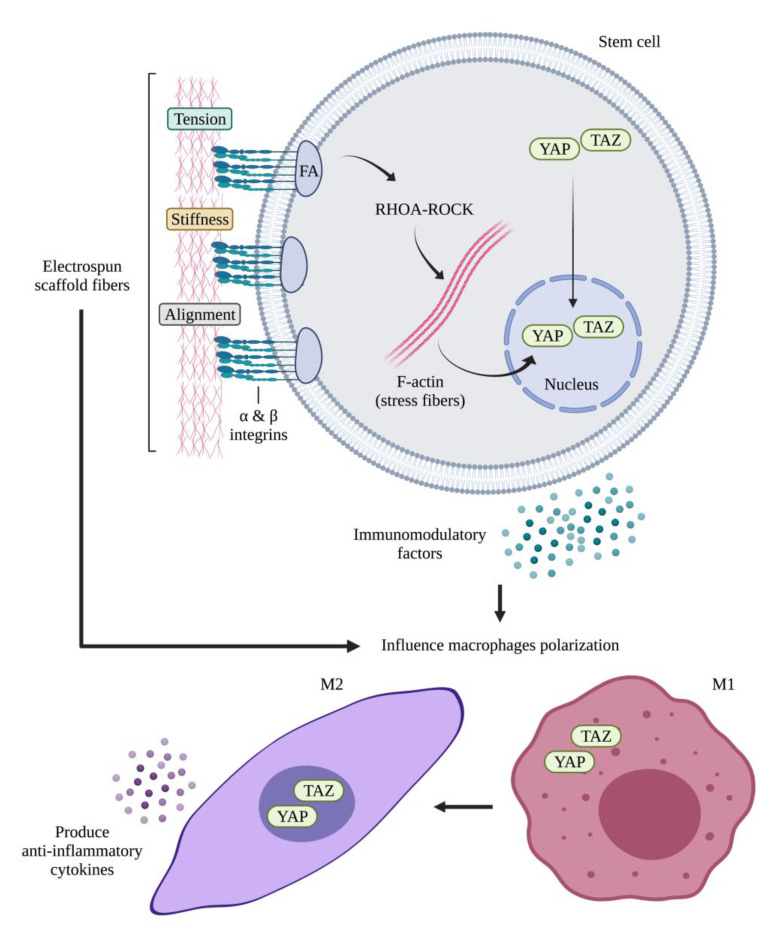
The effect of mechanotransduction cues on RhoA pathway, which in turn act on YAP/TAZ cellular distribution, and their influence on stem and immune cell immunomodulation.

As discussed in the previous paragraphs, the immune induction on stem and immune cells is driven by several factors. Scaffold’s stiffness, porosity, surface hydrophilicity and charge, and fibers’ alignment are all cells’ immunomodulator effectors (Figure 6). Indeed, cells and tissues are sensitive to mechanical signals from their microenvironment, which includes not only all components of force, stress, and strain, but also substrate rigidity, topography, and adhesiveness [209]. Therefore, exploiting this cell’s mechanosensitivity, biomaterials and the resulting biophysical immunomodulatory cues could modulate inflammatory pathways as well influence cells’ activity. This mechanism is called mechanotransduction and represents the ability of cells to react to mechanical cues transforming a physical stimulus in a biological response, which is a critical component of musculoskeletal tissue growth, homeostasis, healing, and degeneration [209,210,211,212]. The aforesaid environmental changes can influence physiological mechanisms at the genetic, cellular, and systemic levels [213]. Speaking of which, the understanding of the mechanosensitivity of tissues and cells represents a promising direction to improve tendon TE.

Indeed, the tendon is a mechanosensitive tissue; this sensitivity, for instance, permits mechanical loading-based therapies. Cells in the tendon are responsible for this adaptive response. For example, the responsiveness of tendon fibroblasts to mechanical loading has been well studied both in vitro and in vivo [210,211,212,214,215,216,217,218,219,220,221,222,223,224]. This has been employed in TE, and several studies have demonstrated that the application of mechanical stimuli on tendon biomimetic scaffolds engineered with stem cells leads to their differentiation towards the tenogenic lineage in a more effective way compared to cells cultured under static conditions [225]. Moreover, mechanical stimuli represent not only a booster of tenogenic differentiation, but they also affect macrophages’ behavior. In this sense, Schoenenberger et al. [93] explored macrophage activation and human macrophage–human tendon fibroblasts’ (hTFs) crosstalk through the cooperative action of intrinsic topological cues from PCL scaffolds (random or aligned fibers) and extrinsic mechanical stimuli. The authors conducted in vitro and in vivo experiments using aligned or randomly oriented PCL constructs in both mechanically loaded and unloaded conditions. The random fiber topography promoted a pro-inflammatory behavior in macrophages and hTFs compared to aligned fibers. Moreover, extrinsic mechanical loading was found to strongly reduce macrophages’ pro-inflammatory markers both in vitro and in vivo. Even more, Schoenenberger et al. found that mechanical co-culture of macrophages and hTFs resulted in a decrease in the CCR7 M1-marker, and increased mechanical loading resulted in an increase in the M2 macrophage phenotype [93].

Therefore, the results of Schoenenberger et al. revealed that macrophage polarization in response to biophysical cues is context based. Moreover, mechanosensitive response of macrophages to biophysical signals generally overshadowed that of tendon fibroblasts, with leading effects of crosstalk between these cell types observed in mechanical co-culture models. Thus, these findings have highlighted a probable role for macrophages as key mechano-sensitive cells that modulate tendon healing, and provide insights into how biological response might be therapeutically modulated by rational biomaterial designs that address the biomechanical niche of recruited cells [93]. However, for improved tendon regeneration, load must be applied with caution [211]. In general, there must be a delicate balance struck between under stimulating and overloading the healing tendon-to-bone interface. Several studies showed that removing all load from the healing site is dangerous [226,227], but unnecessary load is also harmful [228]. This discovery may also apply to TE mechanical loading applications [211]. During dynamic loading of the tissue in culture, an even distribution of force throughout the entire sample is critical, otherwise tissue integrity is compromised by overloading the mechanical connection regions and/or by inhomogeneous mechanical stimulation [225]. Moreover, as demonstrated by Ballotta et al., an overloading of macrophages seeded on PCL electrospun scaffolds caused their polarization to pro-inflammatory M1 phenotype instead of pro-regenerative M2 phenotype [229].

However, how are these mechanical cues converted into cascades of cellular and molecular events that are able to instruct cells’ biology and enhance their immunomodulation? To better understand the mechanotransduction mechanisms, the cellular components that are involved in the transduction of mechanical forces are briefly reviewed.

The cellular membrane, which comes into direct contact with the ECM, is the primary site of force transmission to the cell [209]. Mechanical deformations in the ECM can be transmitted to the actin cytoskeleton which controls cell shape and cell motility and is involved in several cellular functions. The cytoskeleton is composed of microfilaments, microtubules, and intermediate filaments. Microfilaments are actin polymers that bind virtually all the intracellular structures in a continuous, dynamic manner [212]. The ECM’s forces on a cell are in equilibrium with the cell’s forces, and these forces are transmitted through focal adhesion (FA) sites, integrins, cellular junctions, and the ECM. Changes in the cytoskeleton caused by mechanical forces will set off complex signal transduction cascades within the cell by activating integrins and stimulating G protein receptors, receptor tyrosine kinases (RTKs), and MAPKs [212]. Integrins are transmembrane protein heterodimers composed of α- and β-subunits and with three domains: ECM, single transmembrane, and a cytoplasmic domain. The integrin’s ECM domain binds to substrates, while its cytoplasmic domain connects various intracellular proteins that include the cytoskeleton and multiple kinases, including focal adhesion kinase (FAK). Mechanical forces induce integrin conformational activity in cells and enhance cell binding to the ECM. G proteins are made up of a, b, and g subunits that pair membrane receptors and initiate intracellular signaling cascades [212]. The g subunit of heterodimeric G proteins has been detected at integrin-rich focal adhesion sites and adjacent to F-actin stress fibers (SFs), packs of actin filaments and non-muscle myosin II, as well as in other crosslinking proteins [212,230,231]. RTKs are a kind of cell membrane protein that are phosphorylated when stretched or sheared. The MAPK is a protein that travels into the nucleus and interacts with transcription factors and promoters to change gene expression, as well as the ribosomal S6 kinase (RSK) to initiate translation [212,232].

In this brief review of the cellular components involved in the transduction of mechanical forces, FA have a central role. Cells in culture form FA, which are sites of tight adhesions to the underlying ECM. FA are multi-protein integrin-containing structures that cross the plasma membrane and serve as a structural link between the actin cytoskeleton and the ECM, as well as signal transduction regions involved in growth regulation [233]. In detail, FA are formed by the assembly of transmembrane proteins (integrins) that interact with ECM components, such as fibronectin, vitronectin, collagens, and laminins. The extracellular subdomains of the integrin subunits contact the ECM, while the cytoplasmic tail interacts with cytoskeletal actin through several docking proteins such as vasodilator-stimulated phosphoprotein (VASP), paxillin, tyrosin kinase Src and FAK [209,231]. FAK is one of the first molecules recruited to form FA in response to external mechanical stimuli [209]. Its activation by autophosphorylation is considered as the trigger for intracellular mechanotransduction, as it activates downstream mechanotransducers in the cytoplasm. Moreover, the activation of FAK can result in increased cell proliferation through the activation of the MAPK family member extracellular signal-regulated kinases (ERKs) [231]. Cytoskeletal contraction, cell spreading, and other downstream signals reinforce FAK activation in a positive loop, so exogenous force can increase FAK phosphorylation. Moreover, this interaction between FAK and the contractile cytoskeletal network is controlled in the cell in order to maintain tension at key cell sites and manage force transfer to the nucleus [209].

FA are dynamic structures that expand and contract in size because of protein recruitment and disassembly in response to mechanical forces [231]. Their assembly is regulated by the GTP-binding protein Rho [233]. Rho GTPases are small GTPases, members of the Ras superfamily, that work as molecular switches by binding to guanosine triphosphate (GTP) and guanosine diphosphate (GDP). Many cellular processes have been stated to be controlled by them, including actin cytoskeleton remodeling, transcription, cell growth and proliferation, cell motility, morphology, and cell cycle progression. So far, more than 20 members of the Rho GTPase family have been discovered. RhoA, Cdc42, and Rac1 have received the most attention [231].

Tension and myosin are two significant contributors in the development and maturation of SFs and FA. New focal complexes form at the lamellipodia (a cytoskeletal protein actin projection) and are primarily regulated by Rac1 and Cdc42. Mechanical force facilitates the maturation of emerging focal complexes into focal adhesions by recruiting additional proteins and promoting actin polymerization. Previous research has shown that RhoA activity is needed for the formation of SF and FA [231,234]. Moreover, by using myosin inhibitors it has been shown that inhibiting RhoA-mediated myosin activity resulted in a failure to form SFs and FA [231,235]. Current knowledge suggests that mechanical tension activates RhoA signaling pathways and also exposes the binding sites in the mechanosensors [231]. Activated RhoA promotes contractility, which results in isometric tension in cells that are tightly adhered to the substrate [233]. In detail, RhoA activate ROCK, a RhoA effector, which in turn phosphorylates myosin II, which feeds back positively to increase cellular tension [231]. Moreover, ROCK causes actin filament bundling and integrin aggregation in the membrane plane. This last element activates the FAK, resulting in the formation of a multicomponent signaling complex [233]. Some mechanosensor proteins, such as talin, can also undergo conformational changes because of increased stress. Talin stretching exposes new binding sites for the recruitment of other focal adhesion proteins such as vinculin, one of the key components of FA inner core [209,231]. The stretch-sensitive adaptor protein p130Cas is another mechanosensor that contains SH3 domains by which it interacts with vinculin and FAK at the FA site. When mechanically stretched, p130Cas exposes buried tyrosine residues that can be phosphorylated by Src kinase [209,231].

Topography has demonstrated to influence the arrangement of integrins and the formation of FA [231]. Because of the nanometer-sized range of integrins, cells can distinguish topographic changes down to the nanometer scale. During initial adhesion to the microenvironment, cells probe and migrate along the surface using membrane protrusions such as lamellipodia and filopodia as contact guidance. Topographical cues generate mechanical forces that are transmitted into the nucleus via integrins that are linked to the cytoskeleton, according to growing evidence. Topographical cues, on the other hand, may produce mechanical forces that are exerted by FA, activating focal adhesion signaling pathways through FAK and focal adhesion-associated proteins [231]. As previously seen, stiffness represents a topographic feature of scaffold, able to module cells’ immune propriety. Stiffness of the substrate influences integrin clustering, as well as FA assembly and turnover. Cells grown on stiff substrates exhibit elevated intracellular tension, which is distinguished by the presence of stress fibers. The cell-generated contractile force is fought by the stiffness of the ECM, resulting in increased force at the cell–matrix interface that further enhances FA assembly. Consequently, cells grown on stiffer substrates have more focal adhesions [231]. Patel et al., investigated the role of mechanosensitivity in macrophage function, finding that macrophage elasticity (elastic modulus), which is mediated by substrate stiffness, is actively dependent on actin polymerization and RhoGTPase activity. On stiff (150 kPa) polyacrylamide substrates, RAW 264.7 cells (monocyte/macrophage-like cells derived from an Abelson leukemia virus-transformed BALB/c mouse cell line) exhibited organized actin filaments and filopodial projections. However, when treated with a Rho-GTPase inhibitor (C. Difficile toxin), cells resembled those on softer (1.2 kPa) substrates, with an absence of organized actin fibers in projections. Moreover, the elasticity and phagocytic capacity of cells cultured on stiff substrates were also significantly higher, implying that substrate elasticity modulates macrophage elasticity and phagocytosis through actin polymerization [236]. Moreover, using mouse BMMΦs, Beningo and Wang investigated the effects of substrate stiffness on macrophage function. They found that stiff polyacrylamide particles were phagocytized preferentially over soft polyacrylamide particles of equal chemistry via a Rac-1 mediated mechanosensory pathway [237].

In response to mechanical perturbations, mechanical homeostasis in cells is maintained by modifying focal adhesion ligand affinity and by regulating focal adhesion assembly and disassembly. Therefore, mechanical stimuli applied to stem cell engineered scaffold in vitro could cause an upregulation of focal adhesion components [238]. Then, the mechanical information is propagated at the cytoskeleton level, where it affects proteins residing at the membrane or in the cytoplasm, causing structural modification and their shuttling to the nucleus [209]. One of the mediators between the mechanical stimuli sensed by the cytoskeleton and the associated cell response are Yorkie-homologues YAP (Yes-associated protein) and TAZ (transcriptional coactivator with PDZ-binding motif, also known as WWTR1), transcriptional co-activators being the downstream effectors of Hippo pathway [33,239,240]. The activity of YAP and TAZ is important for the growth of entire organs, the amplification of tissue-specific progenitor cells during tissue renewal and regeneration, and cell proliferation. Mechanical signals represent a second aspect of the YAP/TAZ function [241]. The mechanotransduction role of YAP is related to its ability to promote the transcription of genes involved in cell–matrix interaction, ECM composition, and cytoskeleton integrity [209]. YAP/TAZ represent a new class of shuttling proteins that function as mechanotransducers by going back and forth from the nucleus [209]. Since activation of YAP and TAZ results in their accumulation in the nucleus, a significant layer of control of YAP and TAZ occurs at the level of their subcellular distribution. YAP/TAZ are mostly cytoplasmic, but when activated, they shuttle from the cytoplasm to the nucleus to regulate gene expression [84,242,243]. Cell structure, the rigidity and topology of the ECM substrate, and shear stress all influence this cellular distribution. YAP and TAZ, for example, are found in the cytoplasm of cells with low levels of mechanical signaling, such as rounded cells connected to a soft ECM. On the other hand, they are nuclear in cells that experience elevated levels of mechanical signaling, such as cells cultured on stiff substrates or cells that experience deformation and cytoskeletal stress [239,242,244,245]. The mechanosensitive role of YAP and TAZ also results from their interaction with the mechanosensory systems described previously, such as integrins, adaptor proteins such as vinculin and talins, FAK, and SRC-family kinases. For example, in some cell types, inhibitors of non-muscle myosin II or ROCK can direct YAP activation by a stiff ECM. Furthermore, Rho signaling is needed for YAP and TAZ action, which has been experimentally exploited in a variety of systems, either genetically or using Rho inhibitors [239,242,244,245,246,247]. Then, YAP and TAZ serve as nuclear relays for mechanical signals induced by ECM rigidity and cell structure. Indeed, for example ECM stiffness or cell geometry influence their activity. This control includes Rho GTPase activity and actomyosin cytoskeleton stress but is not contingent on the Hippo cascade, it is a parallel and independent pathway (Figure 6) [239].

Moreover, YAP and TAZ were founded as binding proteins for Smads, a key transducer of the TGF-β [159,248,249,250]. Cytoplasmic YAP/TAZ interact with SMAD2/3 through the TAZ coiled-coil domain and participate in Smad2/3 cytoplasmic retaining, even overruling the effects of high levels of TGF-β ligands [249,250]. Moreover, YAP binds SMAD7, enhancing its inhibitory activity against the TGF-β receptors [241]. SMAD2/3 were investigated together with ERK1/2 pathways by Liu et al., Indeed, they explored the adhesion and proliferation of rabbit tenocytes and fibroblasts on multi-layered electrospun PCL–amnion nanofibrous membranes. Liu et al., scaffolds resulted in ERK1/2 and SMAD2/3 phosphorylation upregulation, adhesion and proliferation of tenocytes, fibroblast promotion, and collagen synthesis increase [251].

Quite recently, Tomás et al., investigated the magneto-mechanical stimulation of hASCs seeded on an electrospun PCL fibrous aligned scaffold functionalized with hybrids of cellulose nanocrystals decorated with magnetic nanoparticles. Thus, they evaluated the expression of YAP/TAZ in hASCs cultured for both 11 and 21 days under magneto-mechanical stimulation or in static conditions. Immunofluorescence images of hASCs cultured for 11 days under both conditions showed a predominant YAP/TAZ nuclear expression. The ability of the scaffolds to cause YAP/TAZ activation under both conditions shows that their aligned topography resulted in sufficient cell cytoskeleton stimulation. However, the nuclear to cytoplasmatic YAP/TAZ ratio in stimulated cells at day 11 were significantly higher than the static culture condition. On the other hand, immunofluorescence images of hASCs cultured for 21 days revealed a predominance of cytoplasmatic YAP/TAZ, most likely due to cell crowding and a decline in proliferation [33]. The findings of Tomás et al. match perfectly with the reports of Wan et al. Indeed, they found that both FAK and YAP/TAZ signaling are necessary mechanotransduction pathways through which aligned fibers stimulate the immunomodulatory role of ASCs, whose paracrine secretions can induce M2 phenotypic changes in macrophages [84]. In detail, they selected a PLLA electrospun fibrous scaffold with two different orientations (random vs. aligned) to investigate the effects of fiber orientation on the secretory immunomodulatory behavior of human ASCs. Then, Wan et al. used the conditioned media from ASCs cultured on aligned fiber (for 48 h) to cultivate human ASCs. To illustrate the involvement of FAK, ERK1/2, and YAP/TAZ in this modulation, small molecular inhibitors, including PF573228, PD98059, and Verteporfin, were added to the medium for 48 h prior to further characterization. ASCs cultured on aligned fibrous matrices secreted a significantly higher number of immunomodulatory factors (COX-2, TSG-6, HGF, TGF-β, MHC-G, IL-1ra, M-CSF and MCP-1) compared to that of ASCs cultured on random fibrous matrices. Moreover, these secreted factors by ASCs on aligned fibrous scaffolds addressed macrophages to an anti-inflammatory M2 phenotype as noticeable by the reduced secretion of pro-inflammatory factors (e.g., IL-1b) and enhanced expression of M2 surface markers (CD163 and CD206). Interestingly, ASCs on aligned fibers expressed a preferential nuclear YAP/TAZ localization (43%) to those on random fibers (9%). On the other hand, elevated cytoplasmic YAP/TAZ staining was observed in ASCs on random fibers (28%) compared to those on aligned fibers (2%). Moreover, Wan et al. found that the enhanced immunomodulatory functions of ASCs on aligned fibrous matrices was stopped by treatment with inhibitors of FAK (PF573228), ERK1/2 (PD98059), and YAP/TAZ (Verteporfin), suggesting that FAK-ERK1/2 and YAP/TAZ signaling are involved in mediating the fiber orientation-induced immunomodulation changes in ASCs (Figure 6) [84].

Thus, mechanotransduction could represent a possible explanation for all the different mechanisms through which the intrinsic and extrinsic properties of the scaffolds can immunomodulate stem and immune cells. Indeed, the understanding of the involved mechanisms could lead to the development of novel immunoengineering strategies to be applied in tendon TE.

## 6. Conclusions

Immunoengineering has been introduced as a key challenge discipline in TE with the aim to immunomodulate the inflammatory response using scaffolds. Indeed, understanding the crosstalk amongst scaffold, stem and immune cells might greatly modulate the immune response through the design of immunoinformed scaffolds, which in turn could improve the regenerative potential of immune and stem cells, hence resolving the inflammatory response and avoiding the formation of fibrotic scar tissue. This can be achieved by stimulating macrophage polarization towards an anti-inflammatory phenotype and by recruiting stem cells within the damage tissue improving hence their immunomodulatory properties. Indeed, controlling the intrinsic characteristics of electrospun scaffolds in terms of fiber topography, fiber diameter, pore size and surface chemistry together with the extrinsic ones including mechanical stimuli and scaffold degradation behavior has offered new insights to improve the scaffolds’ regenerative potential to deal with tendinopathies. It has been demonstrated that fiber alignment with adequate fiber diameter size, surface chemistry and functionalization of electrospun scaffolds with specific bioactive molecules can greatly increase stem and immune cell recruitment and switch their immune response towards a pro-regenerative response. Studies concerning the immunoregenerative strategies applied to tendon TE are still few, as confirmed by the scientometric analysis. Further studies are required to better understand the mechanisms behind tendon regeneration as well as scaffold–immune system interaction, in order to improve the performance of the electrospun scaffolds, hence allowing the development of new immunoinformed scaffolds able to completely regenerate tendon damages.

## Figures and Tables

**Table 1:** Overview of the influence of different electrospun scaffolds properties on immune cells.

Scaffold Properties	Material	Parameters	Immune Response	Reference
Diameter size	PDO	Different fiber diameter size (0.35, 2.20, and 2.80 µm)	Increasing fiber diameter → ↑ M2 macrophages expression	[90]
	PCL	Different fiber diameters (0.69 and 5.59 μm)	Increased fiber diameter size (5.59 µm) → ↑ M2 macrophages expression	[91]
Alignment	PCL	Random and aligned fiber orientation; scaffolds unmodified or extended to macro-scale thicknesses of 3 or 10 mm	Expanded scaffolds ↑ regenerative answer and thinner collagen fibrous capsule compared to unexpanded nanofiber scaffolds Aligned fibers (expanded to 3 mm) fewest ↓ number of giant cells	[92]
	PLLA	Five different types of scaffolds: aligned microfibers, aligned nanofibers, random microfibers, random nanofibers, and on film	Nanofibrous PLLA scaffolds ↓ inflammatory response than films and microfibrous scaffolds PLLA film ↑ number of foreign body giant cells than the micro and nanofibrous scaffolds	[29]
	PCL	Random and aligned fiber orientation	Random fibers ↑ pro-inflammatory response compared to aligned fibers	[93]
	PCL	Random and aligned fiber orientation	Aligned fibers → least amount of monocyte adhesion with a thinner fibrous capsule and more fibroblasts infiltration compared to randomly oriented fibers	[94]
Pore size	PDO	Different pore size (0.96, 10.57, and 14.73 µm)	14.73 µm pore size → M2 macrophage polarization, ↑Arginase I and ↓iNOS	[90]
Mechanical stimulus	PCL	7 and 12% cyclic uniaxial strains (0.8 Hz)	7% mechanical strain → ↑ MCP-1, IL-6, IL-10, and MMP-9 (M2 markers) 12% strain → M1 proinflammatory phenotype	[95]
	CE-UPy-PCL	Cyclic strains: 0%, 8% and 14% strain at 0.8 Hz	High strains addressed a pro-inflammatory condition	[96]
	PCL	Static culture (1% constant strain) and dynamic loading (7% cyclic strain at 1 Hz)	Dynamic loading → ↑ CCR7 (M1 marker)	[93]
Surface modification	PLLA	Lubricating layer of chitosan collagen and alginate hydrogel	↓ Protein adsorption	[97]
	PLGA	CTS layer coating	↓ inflammatory cells recruitment and FBGCs formation	[98]
	PLLA	Two layers of PLLA membranes combined into a single layer	↓ Adhesion to the tissues	[99]

PCL: polycaprolactone; CE-UPy-PCL: ureido-pyrimidinone (UPy)-modified Chain Extended Polycaprolactone; PLLA: poly(L-lactide); PDO: polydioxanone; PLGA: poly(lactic-co-glycolic acid); CTS: chitosan; ↑: increase; ↓: decrease.

**Table 2:** Overview of the influence of different electrospun scaffolds properties on the immunomodulatory properties of stem cells.

Material	Stem Cell Type	Propriety	Outcomes	Reference
PCL	Rat ADMSCs	Randomly oriented, aligned and mesh-like electrospun fibers	↑ gene expression of PGE_2_, iNOS, and VEGF within ADMSCs engineered within aligned and mesh-like fibers	[83]
PLLA	Human ADMSCs	Randomly oriented and highly aligned electrospun fibers	↑ gene expression of COX-2, TGF-β, TSG-6, and M-CSF in ADMSCs cultured within aligned fibers ↑ protein expression of COX-2 and TSG-6 and ↑ secreted levels of PGE_2_ in ADMSCs on aligned fibers	[84]
PLGA	Ovine AECs	Electrospun PLGA scaffolds with two different diameter size (1.27 and 2.50 µm)	↑ gene expression of IL-4 and IL-10 and ↓ gene expression of IL-12 and IL-6 within small fiber diameter size (1.27 µm)	[18]
PCL	Human ADMSCs	Electrospun PCL-DT-NPs yarns cultivated under static and magnetic stimulation conditions	↑ gene expression of MMP-1, MMP-2, MMP-3, TIMPs, IL-10, and IL-4 with ↓ gene expression of IL-6 and COX-2 under magnetic stimulation condition	[33]

PCL: polycaprolactone; PLLA: poly(L-lactide); PLGA: poly(lactic-co-glycolic acid); ADMSC: adipose-derived mesenchymal stem cells; AECs: amniotic epithelial stem cells; ↑: increase; ↓: decrease.

**Table 3:** Overview of the influence of bioactive molecules on the immunomodulatory properties of tenocytes, stem and immune cells.

Bioactive Molecule	Scaffold Material	Cell Type	Outcomes in the Studied Cell Type	Reference
NSAIDs	PELA	Macrophages	↓ inflammatory response and ↓TA	[85]
IL-4	CE-UPy-PCL	Macrophages	↑ IL-10, TGF-β1 and MMP-9 ↓ IL-6	[2]
IL-4	PCL	Macrophages	↑M2 macrophage markers (Arginase I, CD206…)	[153]
25-hydroxyvitamin D_3_	PCL	Macrophages	↓ TNF-a, IL-6 and ↑ IL-4, IL-10	[154]
Ibuprofen	PLA	Macrophages	↓TNF-α expression and collagen III deposition	[155]
Mesenchymal stromal exosomes	PEF	BM Macrophages	↑ CD206+ M2 macrophages and the concentration of IL-4, IL-10 and IL13 ↓ concentration of TNF-α and IFN-γ	[156]
IL-4 plasmid-loaded liposomes (aL/p)	MSaP	BM Macrophages	↑ levels of IL-10 and TGF-β ↓ levels of IL-1 and TNF-α	[157]
Melatonin	PCL	Human BMSCs	Inhibition of macrophage (CD68-positive cell) accumulation at the tendon-to-bone interface.	[158]
MSCs-derived ECM	PCL/SF	Human BMSCs	In vitro: ↑ M2 macrophage polarization and ↓ IL -1β, IL-6, CXCL11, IL-10, IL-1R2 and TGF-β1 In vivo: ↓ FBR, thinner fibrotic capsule formation and ↑ M2 macrophage polarization	[159]
bFGF	PLLA	Human vaginal fibroblasts	↑ concentration of TGF-β1 and ↓ concentration of TGF-β1 and ↓ concentration of TNF-α	[160]
IFN-γ	SF/PLGA	Human BMSCs	↑ transcription levels of COX-2 and IDO ↓ transcription levels of TNF-α	[161]
OLE	PHA	Human HaCaTs	↓ concentration of IL-1, IL-6, IL-8 and TNF-α	[162]
IFN-γ and TNF-α	No scaffold	Human BMSCs	↑ gene expression of IDO, iNOS, IL-6, COX-2 and VCAM-1	[163]
PRP	No scaffold	Human tenocytes	↑ concentration of VEGF, RANTES and HGF ↓ gene expression of IL-6, IL-6R, and IL-8	[164]
HGF	No scaffold	Tendon fibroblasts	↑ concentration of MMP-2 and MMP-9, α-SMA, TIMP-1, VEGF and IL-10 ↓ gene expression of IL-6	[165]
BMP-12	No scaffold	Human ASCs	↑ concentration of VEGF, MMP1, MMP8 and IL6	[166]

PCL: polycaprolactone; CE-UPy-PCL: ureido-pyrimidinone (UPy)-modified chain extended polycaprolactone; PLLA: poly(L-lactide); PLA: polylactide; PLGA: poly(lactic-co-glycolic acid); PELA: poly(lactic acid-co-Ethylene glycol-co-Lactic Acid); PEF: fibrous polyester; MsaP: microsol electrospun fiber scaffold; BMMΦs: bone marrow macrophages; BMSCs: bone marrow mesenchymal stem cells; HaCaT: human dermal keratinocytes; bFGF: basic fibroblast growth factor; OLE: olive leaf extract; PRP: platelet-rich plasma; HGF: hepatocyte growth factor; ↑: increase; ↓: decrease.

## Data Availability

Not applicable.
